# Biological age acceleration, genetic susceptibility, and incident diabetic retinopathy risk: A prospective cohort study

**DOI:** 10.1016/j.jnha.2026.100863

**Published:** 2026-05-06

**Authors:** Yuxuan Wang, Lifeng Wang

**Affiliations:** a Department of Ophthalmology, The Affiliated Hospital of Qingdao University, China; b Department of the Colorectal Anal Surgery, The Affiliated Taian City Central Hospital of Qingdao University, China

**Keywords:** Diabetic retinopathy, Biological age acceleration, Polygenic risk score, Genetic susceptibility, UK Biobank

## Abstract

**Background:**

Diabetic retinopathy (DR) remains a major microvascular complication of diabetes, and conventional risk factors do not fully explain individual differences in disease risk. This study investigated the associations of biological age acceleration with incident DR.

**Methods:**

This prospective cohort study included 12,608 UK Biobank participants with diabetes. Three biological age acceleration measures, including phenotypic age acceleration (PhenoAgeAccel), Klemera-Doubal method Biological Age acceleration (KDMAccel), and Gompertz Law-Based Biological Age difference (GOLD BioAgeDiff), were evaluated. Cox proportional hazards models were used to estimate hazard ratios (HRs) and 95% confidence intervals (CIs) for incident DR. Joint effects with polygenic risk score were further assessed. Analyses were also repeated in the general population.

**Results:**

Higher biological age acceleration was consistently associated with an increased risk of incident DR. Per 5-year increase, the fully adjusted HRs were 1.32 (95% CI, 1.26–1.38) for PhenoAgeAccel, 1.13 (95% CI, 1.11–1.16) for KDMAccel, and 1.20 (95% CI, 1.16–1.24) for GOLD BioAgeDiff. Participants with both high genetic risk and accelerated biological aging had the highest risk of DR, and additive interaction was observed between biological age acceleration and genetic susceptibility. Similar patterns were observed in the general population.

**Conclusion:**

The results suggest that accelerated biological aging is associated with a higher risk of incident DR and may have potential relevance for DR risk stratification.

## 1 Introduction

According to the latest International Diabetes Federation Diabetes Atlas, an estimated 589 million adults aged 20–79 years were living with diabetes worldwide in 2024, and this number is projected to rise to 853 million by 2050 [1]. As one of the most common microvascular complications of DM, diabetic retinopathy (DR) imposes a substantial public health burden, particularly given the growing global prevalence of diabetes [2,3]. DR has also been identified as the fifth leading cause of vision impairment and blindness in working-age adults [[4], [5], [6]]. Although several risk factors, including hyperglycemia, hypertension, hyperlipidemia, and longer duration of diabetes, are well established, there is still substantial variability between individuals in the incidence and progression of DR [[7], [8], [9]]. This suggests that conventional risk markers may not fully capture individual susceptibility to DR, highlighting the need for risk stratification markers of DR risk.

There is accumulating evidence to suggest that aging plays a critical role in the development of DR [10]. However, chronological age alone may not adequately capture inter-individual differences in the biological processes underlying aging [11,12]. In this context, biological age has emerged as a promising measure of functional decline across multiple physiological systems [13]. Several biomarker-based biological age algorithms, including phenotypic age (PhenoAge), Klemera–Doubal method Biological Age (KDM-BA), and Gompertz Law-Based Biological Age (GOLD BioAge), have been validated as predictors of morbidity and mortality and may better reflect aging-related risk than chronological age alone [[14], [15], [16]]. These measures have shown predictive value for a range of age-related conditions, including chronic respiratory disease, musculoskeletal disorders, dementia, and stroke [[17], [18], [19], [20]]. However, most previous studies have focused on macrovascular or non-ocular outcomes, whereas prospective evidence regarding biological age acceleration in relation to incident DR, a diabetes-related microvascular complication, remains limited. In addition, whether biological age acceleration and genetic susceptibility jointly contribute to DR risk has not been well established. These gaps highlight the need to clarify the role of biological age acceleration in the development of incident DR.

While accelerated biological aging may contribute to the development of DR, this relationship could be further modulated by genetic susceptibility [21]. Polygenic risk scores (PRS), which aggregate the effects of multiple genetic variants across the genome into a single quantitative metric, providing a way to quantify individual-level genetic predisposition to complex diseases [22]. Although numerous loci associated with DR have been identified through genome-wide association studies (GWAS), the extent to which genetic susceptibility interacts with biological aging in shaping DR risk remains unclear [23]. Understanding such interactions may help to identify subpopulations that are particularly vulnerable, where a combination of accelerated aging and high genetic risk leads to an increased risk of DR.

In the present study, data from the United Kingdom Biobank (UK Biobank) were utilized to evaluate the associations between three widely employed biological age acceleration metrics—PhenoAge acceleration (PhenoAgeAccel), KDM-BA acceleration (KDMAccel), and Gompertz Law-Based Biological Age difference (GOLD BioAgeDiff)—and the risk of incident DR. Furthermore, the interaction between biological age acceleration and genetic susceptibility, as quantified by PRS, was investigated. By focusing on a diabetes-related microvascular complication in a prospective setting, this study aimed to extend current evidence on biological aging beyond previously studied macrovascular and non-ocular outcomes. The robustness of these findings was then evaluated in the general population.

## 2 Methods

### 2.1 Study population

**Fig. 1:**
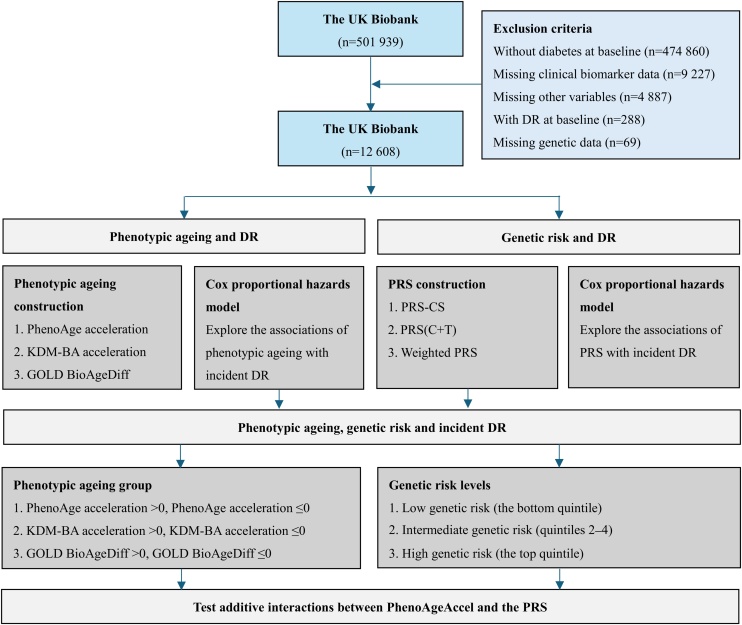
Overview of the study design and analytical process in participants with diabetes. DR: diabetic retinopathy; PhenoAge: Phenotypic age; KDM-BA: Klemera-Doubal method Biological Age; GOLD BioAgeDiff: Gompertz Law-Based Biological Age difference; PRS: polygenic risk score; C + T: clumping and thresholding; CS: continuous shrinkage.

The UK Biobank is a large prospective cohort study comprising over 500,000 individuals recruited across the United Kingdom between 2006 and 2010 [24]. It was designed to investigate the genetic, environmental, and lifestyle determinants of various diseases, with participants undergoing extensive baseline assessments and long-term follow-up. Diabetes at baseline was defined using a previously validated algorithm within the UK Biobank population, incorporating self-reported diagnoses, medication use, and relevant biochemical markers [25]. Specific criteria and the corresponding variables used to define diabetes status are listed in Table S2. From the full cohort (*n* = 501,939), we identified individuals with diabetes and applied the following exclusion criteria: (1) no diabetes diagnosis at baseline (*n* = 474,860); (2) missing clinical biomarker data required for biological age estimation (*n* = 9227); (3) missing covariate data (*n* = 4887); (4) a pre-existing DR diagnosis at baseline (*n* = 288); and (5) missing genetic data (*n* = 69). A complete-case approach was used, and participants with missing biomarker, covariate, or genetic data were excluded before analysis. Following these exclusions, 12,608 participants were included in the primary analysis (Fig. 1). To evaluate the generalizability of our findings, we additionally applied the same inclusion and exclusion criteria to a general population sample (*n* = 271,881), and assessed the associations between biological age acceleration and incident DR (Fig. S1).

### 2.2 Assessment of biological age acceleration

Biological age was assessed using three established algorithms: PhenoAge, KDM-BA, and GOLD BioAge. All three models were constructed using clinical biomarkers. Prior to the calculation of PhenoAge, KDM-BA, and GOLD BioAge, the component biomarkers were winsorized at the 1st and 99th percentiles to minimize the influence of extreme values. PhenoAge was originally developed using a Cox proportional hazards model trained on NHANES data and incorporates nine variables: albumin, C-reactive protein (CRP), creatinine, glucose, lymphocyte percent, white blood cell count (WBC), red cell distribution width (RDW), mean cell volume (MCV), and alkaline phosphatase (ALP) [14]. KDM-BA, based on the Klemera-Doubal algorithm, utilizes nine variables: albumin, systolic blood pressure (SBP), ALP, forced expiratory volume in 1 s (FEV1), creatinine, CRP, HbA1c, total cholesterol, and urea nitrogen [15]. GOLD BioAge was constructed via Lasso-penalized Cox regression using Gompertz mortality modelling and updated specifically for the UKB cohort. It includes ten variables: chronological age, creatinine, glucose, MCV, RDW, albumin, ALP, lymphocyte percent, WBC, and gamma-glutamyl transferase (GGT) [16]. PhenoAgeAccel and KDMAccel were defined as residuals from regressing biological age on chronological age, while GOLD BioAgeDiff was calculated as the difference between GOLD BioAge and chronological age. Detailed equations and included variables for each model are provided in the Supplementary Methods. PhenoAge and KDM-BA were calculated using the BioAge R package. Individuals with values < 0 were classified as "biologically younger", whereas others were considered "biologically older". Furthermore, each biological age acceleration metric was categorized into quartiles (Q1–Q4) based on its distribution, with Q1 representing the lowest acceleration group and Q4 the highest.

### 2.3 Construction of PRS

To quantify individual-level genetic susceptibility to DR, three PRS were constructed using distinct methodologies: PRS-CS (continuous shrinkage), clumping-and-thresholding (C + T) via PRSice-2, and a weighted PRS approach. The weighted PRS was derived from 39 single-nucleotide polymorphisms (SNPs) previously identified in published studies catalogued in the PGS Catalog (Table S1) [23]. Each SNP was weighted by its reported log-odds ratio, and scores were summed to generate a cumulative risk score. For genome-wide approaches, summary statistics from a DR GWAS conducted in individuals of European ancestry in the Million Veteran Program were used as the base dataset [26]. PRS-CS was employed to generate genome-wide PRS using Bayesian regression with continuous shrinkage priors. This method incorporates linkage disequilibrium (LD) information from an external European reference panel (1000 Genomes Project) and estimates SNP effect sizes without relying on arbitrary P-value thresholds [27]. A clumping and thresholding PRS was constructed using PRSice-2, with an LD threshold of r² >0.1 applied within a 1000-kb window [28].

To comprehensively characterize genetic susceptibility to DR, these three PRS approaches were used as complementary methods. Among them, PRS-CS was prioritized as the primary PRS for the joint-effect and interaction analyses because it is a genome-wide Bayesian approach that incorporates LD structure and generally provides robust performance for polygenic prediction [27]. The weighted PRS and C + T PRS were analyzed as complementary approaches to evaluate the consistency of the associations across different PRS-construction strategies.

For the interaction analyses, genetic risk based on each PRS was categorized into low (quintile 1), intermediate (quintiles 2–4), and high (quintile 5) risk groups, following prior literature [29]. All PRS values were standardized (mean = 0, SD = 1) before inclusion in regression models.

### 2.4 Assessment of DR

The primary outcome was incident DR. Diagnoses of DR were ascertained through linked hospital inpatient records from the UK Biobank Hospital Episode Statistics (HES) database, using the following International Classification of Diseases codes: Tenth Revision (ICD-10) code H36.0 and Ninth Revision (ICD-9) code 362.0. Incident DR was defined as the first recorded diagnosis of DR after the baseline assessment. To ensure that only incident cases were included in the analysis, participants with a known diagnosis of DR at or prior to baseline were excluded. The event date was defined as the earliest occurrence of DR recorded in the HES data (Table S2).

### 2.5 Covariates

The covariates were selected based on previous studies and their potential associations with both biological aging and DR [30,31]. Baseline variables included chronological age (years), sex (male or female), ethnicity (White, Mixed, Asian, Black, or Others), education level (degree-level or professional qualification vs. other levels), body mass index (BMI, kg/m²), Townsend Deprivation Index, physical activity (high, moderate, or low), sleep duration (long ≥8 h, moderate 7–8 h, or short ≤6 h), healthy diet (yes or no), smoking status (yes or no), and alcohol consumption (yes or no). Physical activity was categorized as high (≥3000 MET-min/week), moderate (600−3000 MET-min/week), or low (<600 MET-min/week) based on total weekly metabolic equivalent task (MET) minutes [32]. Definitions of smoking, alcohol consumption, and healthy diet were based on previously published studies; detailed variable definitions criteria are provided in Table S2 [33,34].

### 2.6 Statistical analyses

Baseline characteristics were summarized as means ± standard deviations (SD) for continuous variables and counts (%) for categorical variables. Participants with missing data on biomarkers required for biological age estimation, covariates, or genetic information were excluded before analysis. Prior to the calculation of PhenoAge, KDM-BA, and GOLD BioAge, the component biomarkers were winsorized at the 1 st and 99th percentiles to minimize the influence of extreme values. Cox proportional hazards regression models were used to estimate hazard ratios (HRs) and 95% confidence intervals (CIs) for the associations between the three biological age acceleration metrics and the risk of incident DR, with effect estimates expressed per 5-year increase. These metrics were further analyzed as categorical variables in two ways: (1) classified as biologically younger (<0) and biologically older (>0), with the younger group as the reference; and (2) grouped into quartiles (Q1–Q4), using Q1 as the reference. Two multivariable models were constructed: Model 1 adjusted for chronological age, sex, ethnicity, education, BMI, and Townsend deprivation index; Model 2 further adjusted for physical activity, sleep duration, smoking, alcohol consumption, and healthy diet. Other continuous covariates were entered using their original scales. The proportional hazards assumption was assessed using Schoenfeld residuals together with visual inspection of Kaplan–Meier (KM) curves. Restricted cubic spline (RCS) was then used to investigate the non-linear relationship between biological age acceleration metrics and DR risk.

Cox models were also used to assess genetic risk by evaluating the association between each standardized PRS and DR, adjusting for all covariates. PRS values were standardized to a mean of 0 and a standard deviation of 1 before inclusion in regression models. Potential gene–environment interactions were explored by stratifying biological age metrics according to PRS risk categories (low, intermediate, or high). We then calculated the relative excess risk due to interaction (RERI) and the attributable proportion due to interaction (AP) to quantify the additive interaction between biological aging and genetic susceptibility [35]. The 95% CIs for RERI and AP were estimated using 1000 bootstrap samples. Subgroup analyses were conducted stratified by sex, age group, BMI category, smoking status, and physical activity to examine potential effect modification.

To validate the robustness of our findings, all analyses were repeated in the general population. A two-sided P value < 0.05 was considered statistically significant. All statistical analyses were performed using the UKB-RAP.

## 3 Results

### 3.1 Participants

During a median follow-up of 16.1 years, a total of 1430 participants developed DR. The baseline characteristics of the included participants with diabetes are presented in Table 1. The mean chronological age at baseline was 59.32 ± 7.24 years, and 8106 individuals (64.3%) were male. Baseline characteristics of the general population used for sensitivity analysis are provided in Table S3. The distributions of the three biological age acceleration metrics stratified by DR status in both the diabetes cohort and the general population are illustrated in Figs. S2 and S3. Participants with incident DR tended to have higher PhenoAgeAccel, KDMAccel, and GOLD BioAgeDiff than those without incident DR.

**Table 1:** Baseline characteristics among participants with diabetes.

	Total	Without DR	With DR
Chronological age (years)	59.32 ± 7.24	59.23 ± 7.27	60.01 ± 6.99
Sex			
Man	8106 (64.3%)	7149 (64.0%)	957 (66.9%)
Woman	4502 (35.7%)	4029 (36.0%)	473 (33.1%)
Ethnicity			
White	11,307 (89.7%)	10,066 (90.1%)	1241 (86.8%)
Mixed	76 (0.6%)	68 (0.6%)	8 (0.6%)
Asian	639 (5.1%)	543 (4.9%)	96 (6.7%)
Black	298 (2.4%)	251 (2.2%)	47 (3.3%)
Others	288 (2.3%)	250 (2.2%)	38 (2.7%)
Education level			
Degree level or professional education	3482 (27.6%)	3088 (27.6%)	394 (27.6%)
Other levels	9126 (72.4%)	8090 (72.4%)	1036 (72.4%)
BMI (kg/m^2^)	31.01 ± 5.74	30.99 ± 5.73	31.17 ± 5.78
Townsend deprivation index	−0.74 ± 3.31	−0.76 ± 3.30	−0.61 ± 3.37
Physical activity			
Low	3331 (26.4%)	2938 (26.3%)	393 (27.5%)
Moderate	6020 (47.7%)	5364 (48.0%)	656 (45.9%)
High	3257 (25.8%)	2876 (25.7%)	381 (26.6%)
Sleep duration			
Short	3806 (30.2%)	3371 (30.2%)	435 (30.4%)
Moderate	7707 (61.1%)	6856 (61.3%)	851 (59.5%)
Long	1095 (8.7%)	951 (8.5%)	144 (10.1%)
Healthy diet			
No	9954 (78.9%)	8809 (78.8%)	1145 (80.1%)
Yes	2654 (21.1%)	2369 (21.2%)	285 (19.9%)
Smoking			
No	5633 (44.7%)	5014 (44.9%)	619 (43.3%)
Yes	6975 (55.3%)	6164 (55.1%)	811 (56.7%)
Alcohol consumption			
No	8539 (67.7%)	7527 (67.3%)	1012 (70.8%)
Yes	4069 (32.3%)	3651 (32.7%)	418 (29.2%)
White blood cell count (1000 cells/μL)	7.53 ± 1.84	7.53 ± 1.83	7.54 ± 1.87
Red cell distribution width (%)	13.62 ± 0.96	13.61 ± 0.95	13.73 ± 1.03
Mean cell volume (fL)	81.87 ± 5.18	81.91 ± 5.13	81.52 ± 5.51
Lymphocyte percent (%)	28.20 ± 7.35	28.31 ± 7.33	27.40 ± 7.48
C-reactive protein (mg/dL)	0.30 ± 0.38	0.30 ± 0.38	0.31 ± 0.40
Glucose serum (mmol/L)	6.98 ± 2.14	6.86 ± 2.09	7.93 ± 2.28
Albumin (g/L)	45.15 ± 2.73	45.22 ± 2.71	44.58 ± 2.80
Creatinine (mg/dL)	73.96 ± 16.36	73.81 ± 16.11	75.13 ± 18.18
Alkaline phosphatase (U/L)	86.71 ± 24.66	86.33 ± 24.42	89.68 ± 26.29
FEV1 (mL)	2575.07 ± 737.44	2582.67 ± 738.99	2515.72 ± 722.76
Systolic blood pressure (mmHg)	140.52 ± 16.76	140.25 ± 16.70	142.62 ± 17.10
Total cholesterol (mg/dL)	176.16 ± 38.56	176.74 ± 38.70	171.63 ± 37.17
Glycated hemoglobin (%)	6.71 ± 0.87	6.65 ± 0.86	7.18 ± 0.77
Urea nitrogen (mg/dL)	16.02 ± 4.10	15.95 ± 4.05	16.64 ± 4.45
Gamma glutamyl transferase (U/L)	47.36 ± 38.55	47.37 ± 38.45	47.27 ± 39.32
PhenoAge	52.53 ± 9.18	52.22 ± 9.20	54.92 ± 8.68
PhenoAgeAccel	3.25 ± 5.73	3.04 ± 5.65	4.93 ± 6.04
KDM-BA	52.10 ± 13.54	51.62 ± 13.44	55.82 ± 13.74
KDMAccel	3.13 ± 12.45	2.74 ± 12.25	6.11 ± 13.56
GOLD BioAge	61.36 ± 11.04	60.99 ± 11.07	64.24 ± 10.35
GOLD BioAgeDiff	2.04 ± 7.65	1.76 ± 7.59	4.24 ± 7.78

Values are presented as mean ± SD or n (%). FEV1: forced expiratory volume in 1 s; BMI: body mass index; DR: diabetic retinopathy; PhenoAge: phenotypic age; KDM-BA: Klemera-Doubal method Biological Age; GOLD BioAgeDiff: Gompertz Law-Based Biological Age difference; PhenoAgeAccel: phenotypic age acceleration, KDMAccel: Klemera-Doubal method Biological Age acceleration.

### 3.2 Biological age acceleration and incident DR risk

**Fig. 2:**
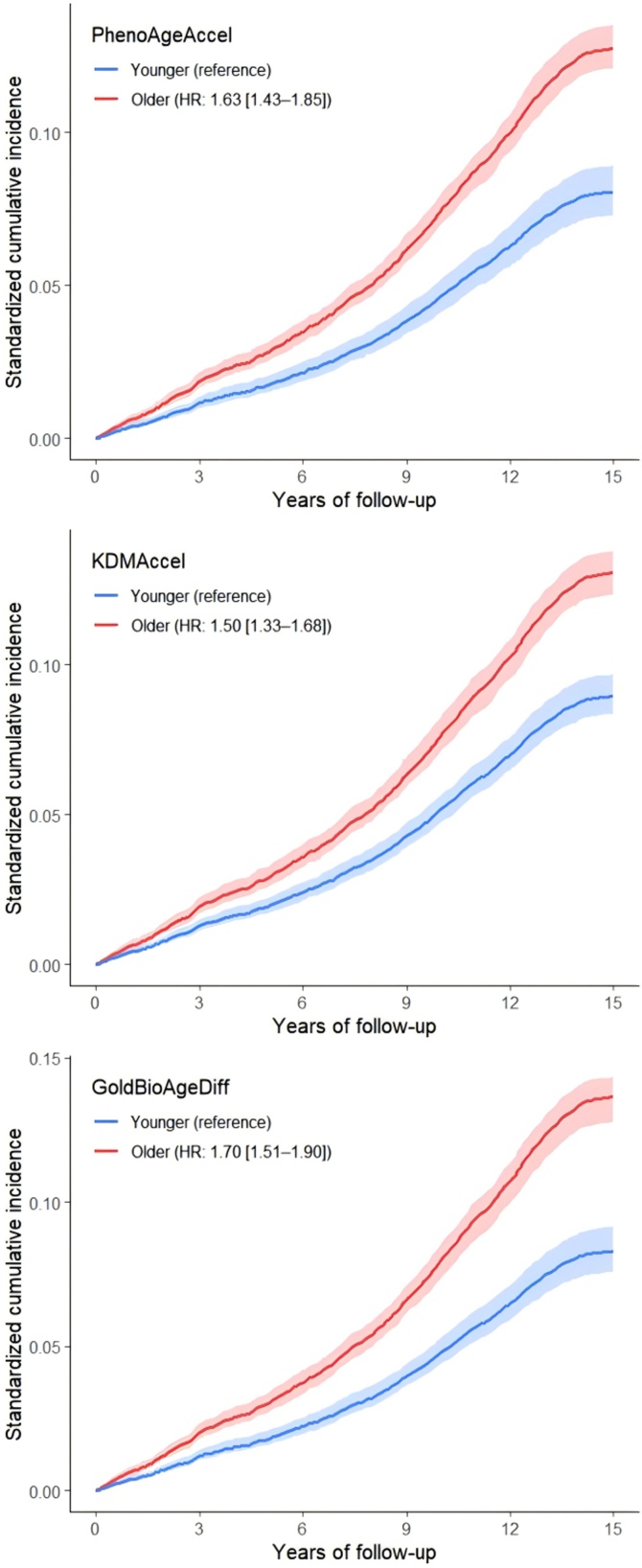
Associations between biological age acceleration and incident DR risk among different biological age groups. Chronological age, sex, ethnicity, education, BMI, and Townsend deprivation index, physical activity, sleep duration, smoking, alcohol consumption, and healthy diet were adjusted in the analyses. PhenoAgeAccel: phenotypic age acceleration, KDMAccel: Klemera-Doubal method Biological Age acceleration; GOLD BioAgeDiff: Gompertz Law-Based Biological Age difference.

**Fig. 3:**
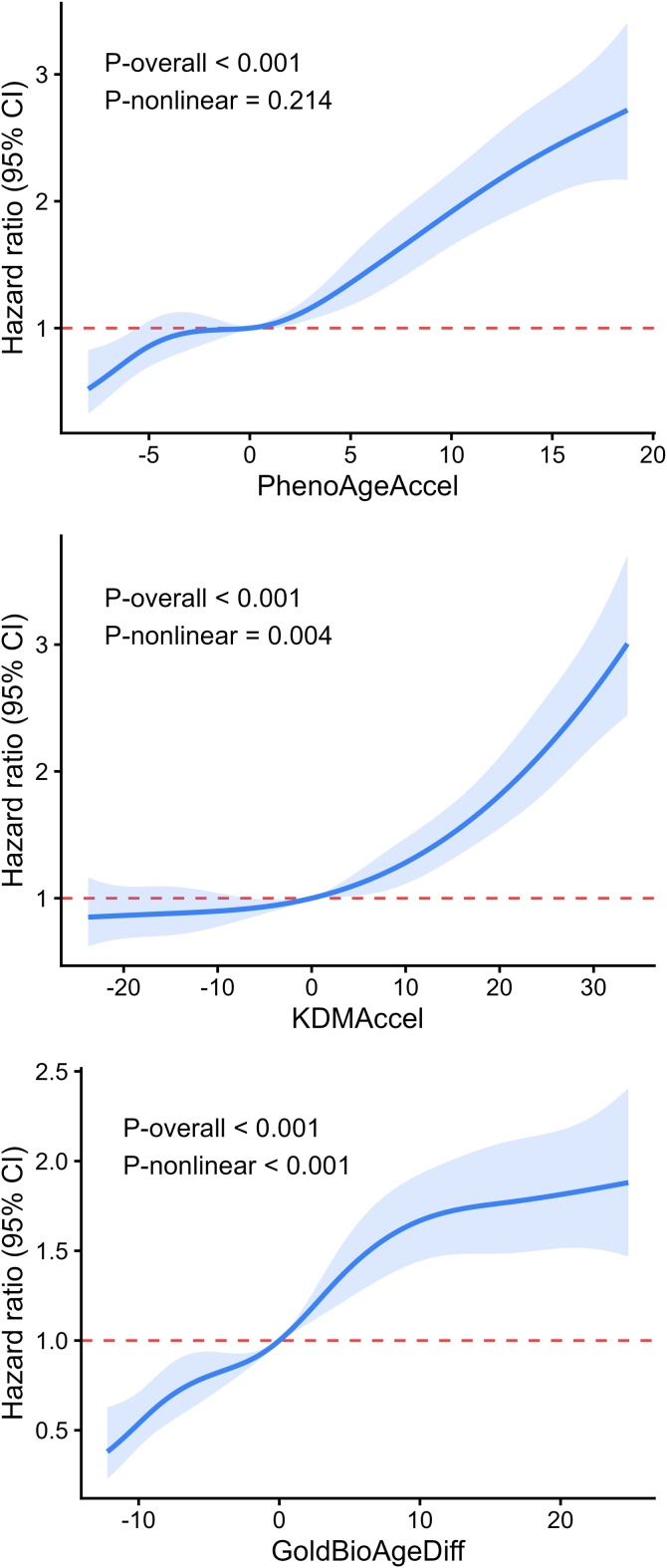
Linear and non-linear association between biological age acceleration and incident DR among participants with diabetes. Chronological age, sex, ethnicity, education, BMI, and Townsend deprivation index, physical activity, sleep duration, smoking, alcohol consumption, and healthy diet were adjusted in the RCS analyses. PhenoAgeAccel: phenotypic age acceleration, KDMAccel: Klemera-Doubal method Biological Age acceleration; GOLD BioAgeDiff: Gompertz Law-Based Biological Age difference.

Higher biological age acceleration was consistently associated with an increased risk of incident DR. In the fully adjusted model, each 5-year increase was associated with a higher risk of DR for PhenoAgeAccel (HR = 1.32, 95% CI: 1.26–1.38), KDMAccel (HR = 1.13, 95% CI: 1.11–1.16), and GOLD BioAgeDiff (HR = 1.20, 95% CI: 1.16–1.24) (Table 2). Similar associations were observed when these measures were analyzed categorically: biologically older individuals had higher DR risk than biologically younger individuals, and a clear dose–response relationship was observed across quartiles, with the highest risk in Q4 (Table 2; Figs. 2 and S4). RCS analyses further supported these associations, showing an approximately linear association for PhenoAgeAccel and nonlinear associations for KDMAccel and GOLD BioAgeDiff (Fig. 3).

**Table 2:** Association between biological age acceleration and DR risk among participants with diabetes.

Exposures	Events/Total	Model 1		Model 2	
		HR (95% CI)	P-value	HR (95% CI)	P-value
PhenoAgeAccel (continuous)					
Per 5 years increase	1430/12,608	1.32 (1.26–1.38)	<0.001	1.32 (1.26–1.38)	<0.001
PhenoAgeAccel (category)					
Biologically younger	308/3815	1.00 (reference)		1.00 (reference)	
Biologically older	1122/8793	1.64 (1.44–1.87)	<0.001	1.63 (1.43–1.85)	<0.001
PhenoAgeAccel (quartile)					
Q1 (<-0.77)	255/3152	1.00 (reference)		1.00 (reference)	
Q2 (-0.77–2.89)	307/3152	1.22 (1.03–1.44)	0.019	1.22 (1.03–1.44)	0.021
Q3 (2.89–6.74)	357/3152	1.47 (1.25–1.73)	<0.001	1.46 (1.24–1.72)	<0.001
Q4 (>6.74)	511/3152	2.20 (1.88–2.57)	<0.001	2.18 (1.86–2.55)	<0.001
KDMAccel (continuous)					
Per 5 years increase	1430/12,608	1.13 (1.11–1.16)	<0.001	1.13 (1.11–1.16)	<0.001
KDMAccel (category)					
Biologically younger	476/5202	1.00 (reference)		1.00 (reference)	
Biologically older	954/7406	1.50 (1.34–1.69)	<0.001	1.50 (1.33–1.68)	<0.001
KDMAccel (quartile)					
Q1 (<-5.45)	286/3152	1.00 (reference)		1.00 (reference)	
Q2 (-5.45–2.74)	309/3152	1.14 (0.97–1.34)	0.126	1.13 (0.96–1.33)	0.138
Q3 (2.74–11.2)	343/3152	1.29 (1.10–1.51)	0.002	1.28 (1.09–1.51)	0.002
Q4 (>11.2)	492/3152	2.03 (1.74–2.38)	<0.001	2.01 (1.72–2.35)	<0.001
GOLD BioAgeDiff (continuous)					
Per 5 years increase	1430/12,608	1.20 (1.16–1.24)	<0.001	1.20 (1.16–1.24)	<0.001
GOLD BioAgeDiff (category)					
Biologically younger	455/5517	1.00 (reference)		1.00 (reference)	
Biologically older	975/7091	1.70 (1.51–1.90)	<0.001	1.70 (1.51–1.90)	<0.001
GOLD BioAgeDiff (quartile)					
Q1 (<-3.24)	229/3152	1.00 (reference)		1.00 (reference)	
Q2 (-3.24–1.14)	307/3152	1.35 (1.13–1.60)	<0.001	1.35 (1.13–1.60)	<0.001
Q3 (1.14–6.26)	390/3152	1.74 (1.47–2.05)	<0.001	1.73 (1.47–2.05)	<0.001
Q4 (>6.26)	504/3152	2.31 (1.96–2.72)	<0.001	2.32 (1.97–2.73)	<0.001

Model 1 adjusted for chronological age, sex, ethnicity, education, BMI, and Townsend deprivation index. Model 2 further adjusted for physical activity, sleep duration, smoking, alcohol consumption, and healthy diet. PhenoAgeAccel: phenotypic age acceleration, KDMAccel: Klemera-Doubal method Biological Age acceleration; GOLD BioAgeDiff: Gompertz Law-Based Biological Age difference.

### 3.3 Subgroup analyses

**Fig. 4:**
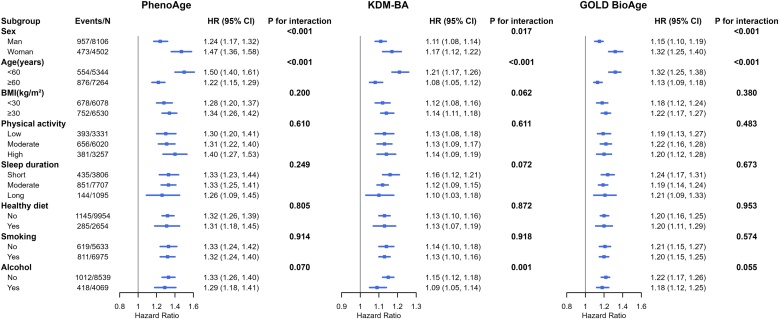
Associations between biological age acceleration and DR risk by different covariates among participants with diabetes. Chronological age, sex, ethnicity, education, BMI, and Townsend deprivation index, physical activity, sleep duration, smoking, alcohol consumption, and healthy diet were adjusted in the subgroup analyses. BMI: body mass index; PhenoAge: phenotypic age; KDM-BA: Klemera-Doubal method Biological Age; GOLD BioAgeDiff: Gompertz Law-Based Biological Age difference; DR: diabetic retinopathy.

Subgroup analyses were performed to assess the associations between biological age acceleration and incident DR across various subgroups (Fig. 4). PhenoAgeAccel was significantly associated with increased DR risk in all subgroups, with a particularly stronger association observed in females (HR: 1.47, 95% CI: 1.36–1.58; P for interaction < 0.001) and participants aged <60 years (HR: 1.50, 95% CI: 1.40–1.61; P for interaction < 0.001). Similarly, KDMAccel and GOLD BioAgeDiff also exhibited consistent associations across all subgroups, with notable effect modification by sex and age. For instance, the association between GOLD BioAgeDiff and DR was stronger in women (P for interaction <0.001) and those younger than 60 years (P for interaction <0.001). No significant interaction was observed for other factors, including BMI, physical activity, sleep duration, healthy diet, or smoking status, suggesting the observed associations were generally robust.

### 3.4 PRS and incident DR risk

Higher genetic risk was also associated with increased incident DR risk. In the fully adjusted model, both PRS-CS and weighted PRS were significantly associated with DR, whereas PRS-C + T was not (Table 3; Fig. S5). Similarly, participants in the high genetic risk group had a higher DR risk than those in the low genetic risk group for PRS-CS and weighted PRS. Among the three approaches, PRS-CS showed the highest predictive performance, further supporting its use as the primary PRS in the joint-effect analyses.

**Table 3:** Association between PRS and DR risk.

Exposures	Events	Model 1		Model 2	
		HR (95% CI)	P-value	HR (95% CI)	P-value
PRS-CS (continuous)					
Per 1 SD increase	1430	1.10 (1.04, 1.16)	<0.001	1.10 (1.04, 1.16)	<0.001
PRS-CS (category)					
Low risk	240	1.00 (reference)		1.00 (reference)	
Intermediate risk	858	1.21 (1.05, 1.39)	0.010	1.21 (1.05, 1.39)	0.010
High genetic risk	332	1.44 (1.22, 1.70)	<0.001	1.44 (1.22, 1.70)	<0.001
PRS-C + T (continuous)					
Per 1 SD increase	1430	1.03 (0.97, 1.08)	0.346	1.02 (0.97, 1.08)	0.358
PRS-C + T (category)					
Low risk	286	1.00 (reference)		1.00 (reference)	
Intermediate risk	836	0.95 (0.83, 1.09)	0.448	0.94 (0.82, 1.08)	0.397
High genetic risk	308	1.05 (0.89, 1.24)	0.551	1.05 (0.89, 1.23)	0.562
Weighted PRS (continuous)					
Per 1 SD increase	1430	1.12 (1.06, 1.18)	<0.001	1.12 (1.06, 1.18)	<0.001
Weighted PRS (category)					
Low risk	243	1.00 (reference)		1.00 (reference)	
Intermediate risk	876	1.24 (1.08, 1.43)	0.003	1.25 (1.08, 1.44)	0.003
High genetic risk	311	1.40 (1.18, 1.65)	<0.001	1.40 (1.18, 1.66)	<0.001

Model 1 adjusted for chronological age, sex, ethnicity, education, BMI, and Townsend deprivation index. Model 2 Further adjusted for physical activity, sleep duration, smoking, alcohol consumption, and healthy diet. C + T: clumping and thresholding; CS: continuous shrinkage; PRS: polygenic risk score.

### 3.5 Joint effects and interactions of biological age acceleration and genetic risk

Joint-effect analyses showed that DR risk increased progressively with the combined presence of greater biological age acceleration and higher genetic risk. Across all three biological age measures, biologically older individuals had higher DR risk than biologically younger individuals within each PRS stratum, and the highest risk was consistently observed among those with both biological age acceleration and high genetic risk (Table 4). Additive interaction analyses further supported a joint effect of biological aging and genetic susceptibility on DR risk (Table S4).

**Table 4:** Joint effects of biological age acceleration and genetic risk on DR risk among participants with diabetes.

PhenoAgeAccel				
Subgroup	Events/Total	Incidence events per 100,000 person-years	HR (95% CI)	P-value
Low genetic risk				
Biological younger	55/758	462.04	1.00 (reference)	
Biological older	185/1764	676.76	1.45 (1.07–1.97)	0.016
Intermediate genetic risk				
Biological younger	181/2299	503.85	1.08 (0.80–1.46)	0.616
Biological older	677/5266	845.77	1.82 (1.38–2.40)	<0.001
High genetic risk				
Biological younger	72/758	611.07	1.33 (0.93–1.88)	0.115
Biological older	260/1763	981.99	2.14 (1.59–2.86)	<0.001
KDMAccel				
Subgroup	Events/Total	Incidence events per 100,000 person-years	HR (95% CI)	P-value
Low genetic risk				
Biological younger	90/1039	554.27	1.00 (reference)	
Biological older	150/1483	652.11	1.22 (0.94–1.59)	0.138
Intermediate genetic risk				
Biological younger	287/3144	589.72	1.06 (0.84–1.35)	0.607
Biological older	571/4421	848.42	1.59 (1.27–2.00)	<0.001
High genetic risk				
Biological younger	99/1019	625.60	1.13 (0.85–1.51)	0.386
Biological older	233/1502	1038.57	1.99 (1.56–2.55)	<0.001
GOLD BioAgeDiff				
Subgroup	Events/Total	Incidence events per 100,000 person-years	HR (95% CI)	P-value
Low genetic risk				
Biological younger	71/1088	414.55	1.00 (reference)	
Biological older	169/1434	764.26	1.80 (1.36–2.38)	<0.001
Intermediate genetic risk				
Biological younger	268/3279	523.89	1.25 (0.96–1.62)	0.095
Biological older	590/4286	910.31	2.15 (1.68–2.75)	<0.001
High genetic risk				
Biological younger	116/1150	652.37	1.58 (1.17–2.12)	0.003
Biological older	216/1371	1054.79	2.50 (1.91–3.27)	<0.001

Chronological age, sex, ethnicity, education, BMI, and Townsend deprivation index, physical activity, sleep duration, smoking, alcohol consumption, and healthy diet were adjusted in the analyses. PhenoAgeAccel: phenotypic age acceleration, KDMAccel: Klemera-Doubal method Biological Age acceleration; GOLD BioAgeDiff: Gompertz Law-Based Biological Age difference; DR: diabetic retinopathy.

### 3.6 Sensitivity analyses

Sensitivity analyses in the general population yielded broadly consistent results. All three biological age acceleration measures remained significantly associated with an increased risk of incident DR, and the associations were generally consistent across continuous, categorical, quartile-based, and subgroup analyses (Table S5; Figs. S6–S8). Similar to the findings in participants with diabetes, stronger associations were observed among women and individuals aged <60 years. Joint analyses with PRS also supported the combined effect of biological age acceleration and genetic susceptibility on DR risk, with the highest risk observed among participants with both accelerated biological aging and high genetic risk (Tables S6 and S7; Fig. S9). Overall, these findings supported the robustness of the main results.

## 4 Discussion

This large, prospective cohort study, which was based on participants with diabetes from the UK Biobank, showed that biological age acceleration, as measured by PhenoAgeAccel, KDMAccel and GOLD BioAgeDiff, was significantly associated with an increased risk of incident DR. These associations persisted across multiple models, including continuous and categorical analyses, quartile-based risk gradients, and non-linear dose–response analyses. The joint effects of biological aging and PRS further amplified the risk of DR, with evidence of additive interaction. An important contribution of the present study is that it extends the investigation of biological age acceleration from previously studied macrovascular or non-ocular outcomes to incident DR, a clinically important diabetes-related microvascular complication.

The pathogenesis of DR is multifactorial and is primarily driven by chronic hyperglycaemia, with additional contributions from inflammation, oxidative stress, metabolic dysregulation, endothelial dysfunction, and microvascular injury [[36], [37], [38], [39]]. Biological aging is likewise characterized by cumulative cellular and molecular damage, including impaired stress responses, mitochondrial dysfunction, epigenetic alterations, and chronic low-grade inflammation [40,41]. These aging-related processes overlap with pathways implicated in DR, suggesting that accelerated biological aging may reflect a systemic vulnerability that increases susceptibility to diabetic microvascular complications [[42], [43], [44], [45]]. However, the biological age measures used in this study were derived from routine systemic biomarkers and were not designed to capture retina-specific pathophysiology. Therefore, although the observed associations are consistent with shared systemic mechanisms relevant to DR, the mechanistic links between individual biological age measures and retinal microvascular biology remain unclear. The present findings should thus be interpreted as supporting biological plausibility at the level of systemic aging-related dysregulation rather than measure-specific retinal mechanisms.

Previous studies have primarily focused on the relationship between chronological age and DR, with few investigating the role of biological aging. A recent cross-sectional analysis based on the National Health and Nutrition Examination Survey (NHANES) 2005–2008, PhenoAge (OR = 1.11, 95% CI: 1.07–1.14) and KDM-BA (OR = 1.12, 95% CI: 1.06–1.18) were significantly associated with DR after adjusting for covariates, whereas chronological age was not [46]. Other studies have also examined the correlation between alternative indicators of biological aging and DR. A case–control study reported that telomere mean length (TML) in peripheral blood monocytes was shorter in diabetic patients with non-proliferative or proliferative DR compared to those without DR and healthy controls. This suggests a potential link between telomere attrition and DR severity [47]. However, another study involving 147 patients with type 2 diabetes found that leukocyte telomere length (LTL) was not associated with an increased prevalence of common diabetic complications, including DR. Nevertheless, shorter LTL was correlated with adverse lipid profiles and glycemic parameters in patients with DR, suggesting that telomere shortening may still reflect metabolic dysregulation related to DR [48]. The exact mechanism still requires further clarification through future research.

Subgroup analyses revealed that the association between accelerated biological aging and incident DR were consistently stronger in women and individuals younger than 60 years. These findings were observed across all three aging measures and remained robust after adjusting covariates, suggesting potential effect modification by sex and age. There are several potential mechanisms that may underlie these observations. First, women may be more susceptible to systemic inflammation and metabolic disturbances associated with biological aging due to hormonal fluctuations, particularly around the time of the menopause [49]. Estrogen deficiency has been linked to increased oxidative stress and vascular vulnerability, which may exacerbate retinal microvascular injury in the presence of accelerated aging [50]. Secondly, accelerated biological aging in younger individuals may indicate an early departure from physiological homeostasis, possibly caused by prolonged exposure to adverse environments or unhealthy lifestyles [51]. Consequently, biological aging may be a more sensitive indicator of early retinal damage in this group, even before traditional risk factors reach clinical thresholds. These findings highlight the importance of incorporating biological aging assessments into personalized DR risk stratification, particularly for younger and female populations, who might otherwise be overlooked by conventional screening strategies [52]. By contrast, no other factors exhibited a consistent effect on the three biological aging indicators.

Compared with genetically low-risk individuals who were biologically younger, those who were biologically older and genetically high-risk had significantly elevated DR risks (HRs: 2.14 for PhenoAgeAccel, 1.99 for KDMAccel, and 2.50 for GOLD BioAgeDiff) across all three measures of biological aging. These findings suggest that there may be an additive interaction between biological aging and genetic susceptibility. One possible explanation is that biological aging may exacerbate the deleterious effects of genetic variants involved in inflammatory, vascular, or metabolic pathways associated with DR [13,53]. Conversely, genetically high-risk individuals who remain biologically younger may have better immune homeostasis and vascular integrity, potentially buffering DR progression. These findings suggest that combined information on biological age acceleration and genetic susceptibility may help identify subgroups at higher risk of DR and may have potential relevance for future risk stratification research.

Although some HRs were modest, they may still be clinically meaningful in the context of DR as a common and progressive complication with substantial long-term burden. The consistent associations across three biological age measures and multiple analytical approaches, together with the stronger joint effects observed in those with high genetic susceptibility, support the potential value of biological age acceleration for risk stratification rather than as a standalone predictor. From a clinical perspective, these findings suggest that combining biological age acceleration with genetic susceptibility may improve the identification of individuals at particularly high risk of DR beyond the use of conventional risk factors alone, and may help identify subgroups who could benefit from closer ophthalmic monitoring, earlier preventive intervention, or more individualized screening intervals.

This study has several important strengths. First, it is based on a large and well-characterized prospective cohort with long-term follow-up and a substantial number of incident DR cases. This enhances both statistical power and the validity of temporal inferences. Secondly, the study systematically evaluated three biological age acceleration metrics: PhenoAgeAccel, KDMAccel, and GOLD BioAgeDiff, all derived from routinely measured clinical biomarkers. This approach enhances the robustness and clinical relevance of our findings. In addition, we incorporated a validated polygenic risk score for DR and examined its joint effect with biological aging, providing new insights into gene–environment interactions in DR development. Moreover, stratified and subgroup analyses allowed us to investigate potential effect modification across demographic and lifestyle factors. The consistent patterns observed across these subgroups further support the generalizability of our results. Lastly, we applied multiple modelling strategies, including continuous, categorical and spline-based approaches, as well as conducting sensitivity analyses in the general population. These approaches collectively reinforce the reliability of our conclusions.

Several limitations should also be acknowledged. First, as this was an observational cohort study, the findings should be interpreted as associations rather than evidence of causality. Biological aging metrics were assessed only at baseline, which limited our ability to capture longitudinal changes or aging trajectories over time. Second, although we adjusted for a broad range of covariates, some clinically important factors for DR were not fully captured. In particular, baseline diabetes was defined using a previously validated UK Biobank algorithm that integrates multiple sources of information rather than a single clearly recorded onset date, which limited our ability to derive diabetes duration consistently for inclusion as a covariate. Moreover, longitudinal data on glycaemic control during follow-up were not sufficiently complete or consistently available for all participants, which limited our ability to reliably adjust for glycaemic control over time. Treatment-related variables were also not comprehensively modelled. Therefore, residual confounding cannot be excluded. Third, the definitions of baseline diabetes and incident DR were based on administrative and coded health-record data, which may have introduced some degree of misclassification bias. Although baseline diabetes was identified using a previously validated UK Biobank algorithm and DR was ascertained using linked hospital inpatient records with ICD-9 and ICD-10 codes, coding inaccuracies, incomplete disease capture, and under-ascertainment of milder cases remain possible. In particular, the DR definition was more likely to capture clinically recognized or relatively severe cases and may have missed milder disease diagnosed in outpatient or screening settings. Therefore, the true incidence of DR may have been underestimated, and our findings may be more generalizable to hospital-recorded DR than to the full spectrum of DR. Fourth, we used a complete-case approach and excluded participants with missing biomarker, covariate, or genetic data. If these excluded individuals differed systematically from those included in the analysis, selection bias may have been introduced. Fifth, the generalizability of our findings may be limited because the UK Biobank cohort is predominantly composed of individuals of European ancestry, which may restrict the applicability of these results to more diverse or higher-risk populations. Finally, although the biological aging indicators used in this study were based on routinely available biomarkers, they may not fully capture other dimensions of aging, such as epigenetic, mitochondrial, or proteomic changes. In addition, these metrics primarily reflect systemic biological aging rather than tissue-specific processes and therefore may not fully reflect the local pathophysiological changes occurring in the retina during DR development.

## 5 Conclusion

This large prospective cohort study showed that accelerated biological aging was associated with a higher risk of incident DR among individuals with diabetes and in the general population. These associations were consistent across three biological age acceleration measures and were stronger among individuals with greater genetic susceptibility. Our findings suggest that biological age acceleration may provide additional phenotypic information relevant to DR risk stratification. However, whether it adds predictive information beyond established clinical risk factors requires further study. Further research is also needed to validate these findings in more diverse populations and to clarify their potential relevance in formal prediction settings.

## CRediT authorship contribution statement

Yuxuan Wang: Writing - Original draft, Formal analysis, Data curation, Project administration;

Lifeng Wang: Writing - Review & Editing, Funds.

## Ethics approval

The data used in this study had previously been approved by the ethics committee (https://www.ukbiobank.ac.uk/).

## Funding

Not applicable.

## Declaration of Generative AI and AI-assisted technologies in the writing process

During the preparation of this manuscript, the authors used DeepL Write to improve the language and readability of the text. No generative AI or AI-assisted technologies were used to generate, alter, or manipulate any scientific content, figures, images, or artwork.

## Availability of data

The data are available from the UK Biobank.

## Declaration of competing interest

The authors declare no competing interests.
