# Supplementary material for: Biological age acceleration, genetic susceptibility, and incident diabetic retinopathy risk: A prospective cohort study
Source: J Nutr Health Aging. 2026 May 6;30(6):100863. doi: 10.1016/j.jnha.2026.100863 (PMC13156640; doi:10.1016/j.jnha.2026.100863)
Supplement: Supplementary file 1 [file mmc1.docx]

**Supplementary file**

Supplementary Methods

Figure S1. Overview of the study design and analytical process in overall population.

Figure S2. Distributions of three biological age acceleration metrics by DR status among participants with diabetes.

Figure S3. Distributions of three biological age acceleration metrics by DR status in overall population.

Figure S4. Associations of biological age acceleration quartiles with incident DR risk among participants with diabetes.

Figure S5. PRS and incident DR risk among participants with diabetes: grouped quintiles (Q1 as reference).

Figure S6. Linear and non-linear association between biological age acceleration and incident DR in overall population.

Figure S7. Associations of biological age acceleration quartiles with incident DR risk in overall population.

Figure S8. Associations between biological age acceleration and DR risk by different covariates in overall population.

Figure S9. PRS and incident DR risk in overall population: grouped quintiles (Q1 as reference).

Table S1. Summary of reported susceptibility loci used to construct the weighted PRS.

Table S2. Coding and definition information of variable in the UK Biobank.

Table S3. Baseline characteristics in overall population.

Table S4. Additive interaction between biological age acceleration and PRS for incident DR among participants with diabetes.

Table S5. Association between biological age acceleration and DR risk in overall population.

Table S6. Joint effects of biological age acceleration and genetic risk on DR risk in overall population.

Table S7. Additive interaction between biological age acceleration and PRS for incident DR in overall population.

**Supplementary methods**

**PhenoAge**

PhenoAge (phenotypic age) was calculated using the Levine et al. composite mortality‐based algorithm, which maps a set of clinical biomarkers to a predicted mortality risk and then transforms that risk onto the age scale. For each participant we first computed the linear predictor

$$\begin{matrix} xb & =-19.907-0.0336\times\text{albumin}+0.0095\times\text{creatinine}+0.1953\times\text{glucose} \\ & +0.0954\times\ln\left( \text{C-reactive protein} \right)-0.012\times\text{lymphocyte \%}+0.0268\times\text{mean corpuscular volume} \\ & +0.3306\times\text{red cell distribution width}+0.00188\times\text{alkaline phosphatase}+0.0554\times\text{white blood cell count} \\ & +0.0804\times\text{chronological age}, \end{matrix}$$

then translated it to 10-year mortality risk using

$$\text{mortality risk}=1-\exp\text{ }[-\exp(xb)(\exp(120\gamma)-1)],\gamma=0.0076927.$$

Finally, PhenoAge was obtained as

$$\text{PhenoAge}=141.50225+\frac{\ln\text{ }[-0.00553\times\ln(1-\text{mortality risk})]}{0.090165}.$$

Biomarkers were measured at baseline and used in their conventional clinical units (albumin, g/dL; creatinine, mg/dL; glucose, mg/dL; C-reactive protein, mg/L—natural log–transformed as specified; lymphocyte percentage, %; mean corpuscular volume, fL; red cell distribution width, %; alkaline phosphatase, U/L; white blood cell count, 10⁹/L). PhenoAge acceleration (PhenoAgeAccel) was defined as the residual from a linear regression of PhenoAge on chronological age (or equivalently PhenoAge − chronological age, when appropriate), with positive values indicating advanced biological aging relative to age peers. Data processing (winsorization, outlier handling, and missingness) followed the procedures described in the Methods.

**KDM-BA**

KDM-BA (Klemera–Doubal method Biological Age) was computed following the Klemera–Doubal method, which estimates an individual’s biological age by regressing chronological age (CA) on multiple biomarkers in a reference sample and then combining the biomarker-specific predictions into a single estimator. In our study the biomarker set comprised nine routinely measured indicators: forced expiratory volume in one second (FEV₁), systolic blood pressure, and seven blood chemistry parameters—albumin, alkaline phosphatase, blood urea nitrogen, creatinine, C-reactive protein, glycated hemoglobin (HbA1c), and total cholesterol. For each biomarker *i*, we fitted a linear model in the reference sample, CA = *kᵢ*·*xᵢ* + *qᵢ* + εᵢ, and obtained the slope (*kᵢ*), intercept (*qᵢ*), and root mean squared error (*sᵢ*). Individual-level KDM-BA was then calculated as

$${\mathrm{KDM}\text{-}\mathrm{BA}}_{\mathrm{EC}}=\frac{\sum_{i=1}^{n} (x_{i}-q_{i})\text{ }\frac{k_{i}}{s_{i}^{2}}\text{ }+\text{ }\frac{\mathrm{CA}}{S_{BA}^{2}}}{\sum_{i=1}^{n} {(\frac{k_{i}}{s_{i}})}^{2}\text{ }+\text{ }\frac{1}{S_{BA}^{2}}},$$

where *xᵢ* is the person’s observed value for biomarker *i*, *n* is the number of biomarkers, and $S_{BA}$is a scaling factor equal to the square root of the variance in CA explained by the biomarker set in the reference sample (used to stabilize the estimator around CA). Higher values indicate older biological age. All biomarkers were taken at baseline; details of quality control and handling of missing data are provided in the main Methods.

**GOLD BioAge**

GOLD BioAge (Gompertz law–based biological age) was derived using a penalized survival framework in which the Gompertz mortality hazard was regressed on a panel of clinical biomarkers and chronological age (CA). Specifically, we fitted a Lasso‐penalized Cox/Gompertz model in the training cohort, using five-fold cross-validation to select the penalty parameter λ; λ_{1se} was chosen to favor a sparse and robust estimator. The final model retained 10 predictors: CA, creatinine, glucose, mean cell volume (MCV), red cell distribution width (RDW), albumin, alkaline phosphatase (ALP), lymphocyte percent (LYM), white blood cell count (WBC), and γ-glutamyl transferase (GGT). GOLD BioAge is computed as a weighted linear combination of these variables:

UK Biobank refit (coefficients re-estimated to account for population differences):

$$\mathrm{GOLD}\text{ }\mathrm{BioAge}\text{ }=\text{ Age}+5.3832\times\text{Creatinine}+1.4168\times\text{Glucose}+0.4206\times\text{MCV}+3.3162\times\text{RDW}-5.0793\times\text{Albumin}+0.0385\times\text{ALP}-0.1899\times\text{LYM}+0.9120\times\text{WBC}+0.1007\times\text{GGT}-78.6519.$$

All biomarkers were measured at baseline using routine clinical assays in their native units. As an aging‐acceleration metric, GoldBioAgeDiff = GOLD BioAge − CA, where positive values indicate biologically older status relative to chronological age.

**Alcohol consumption**

Drinking status was determined using UK Biobank field 1558 (alcohol intake frequency) together with beverage-specific weekly amounts (fields 1568, 1578, 1588, 1598, 1608, 5364). Non-drinkers were participants reporting “never” (code 6) and zero intake across beverages; drinkers were those reporting any frequency 1–5 or a positive intake in any beverage. Responses “do not know” (−1) and “prefer not to answer” (−3) were treated as missing. For dose quantification, weekly units were computed as: wine/champagne = 2 units/glass, beer/cider = 1 unit/pint, spirits = 2 units/measure, fortified/other = 2 units/glass; totals were summed across beverages and divided by 7 for units/day. Optional intensity categories were defined as ≤2 units/day for men and ≤1 unit/day for women (low-risk), otherwise high-risk; alternatively ≤14 vs >14 units/week in sensitivity analyses.

**Healthy diet**

**“**Healthy diet” was defined using a 10-component food-group checklist. Participants were classified as having a healthy diet if ≥5 of the following 10 criteria were met:

1. Fruit (fresh or dried) ≥ 3 servings/day;
2. Vegetables (cooked, salad, or raw) ≥ 3 servings/day;
3. Whole grains (e.g., whole-meal/whole-grain bread, bran, oat, muesli) ≥ 3 servings/day;
4. Fish (oily or non-oily) ≥ 2 servings/day;
5. Dairy (e.g., milk, cheese) ≥ 2 servings/day;
6. Vegetable oils (e.g., olive-oil–based, sunflower/polyunsaturated oil–based, soft margarine, low/reduced-fat spread) ≥ 2 servings/day;
7. Processed meats ≤ 1 serving/day;
8. Unprocessed red meats (poultry, beef, lamb/mutton, pork) ≤ 2 servings/day;
9. Refined grains (white/brown bread, other breads, biscuits, other cereals) ≤ 2 servings/day;
10. No consumption of sugar-sweetened beverages.

**
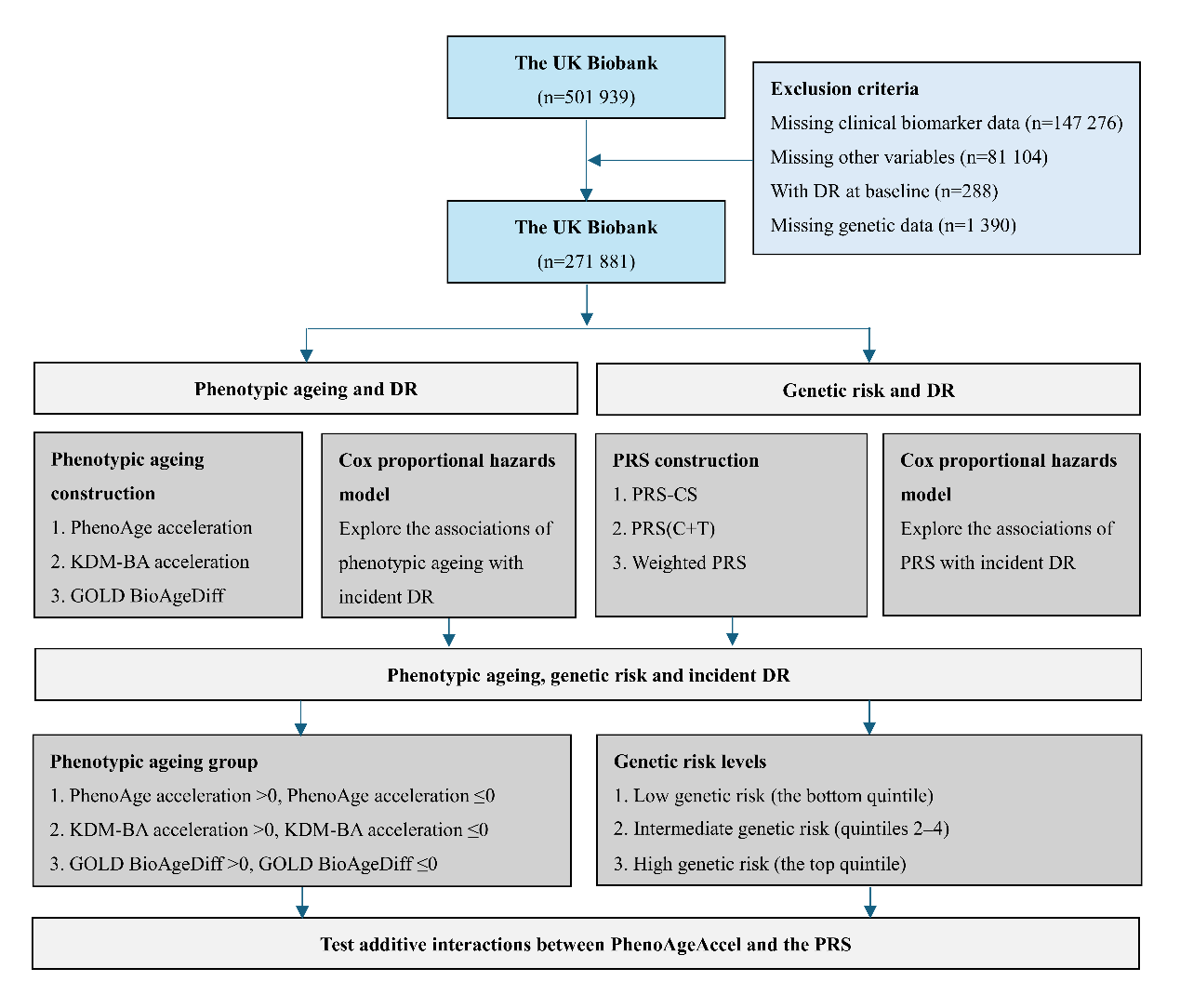
**

Figure S1. Overview of the study design and analytical process in overall population. DR: diabetic retinopathy; PhenoAge: phenotypic age; KDM-BA: Klemera-Doubal method Biological Age; GOLD BioAgeDiff: Gompertz Law-Based Biological Age difference; PRS: polygenic risk score; CS: continuous shrinkage; C+T: clumping and thresholding.


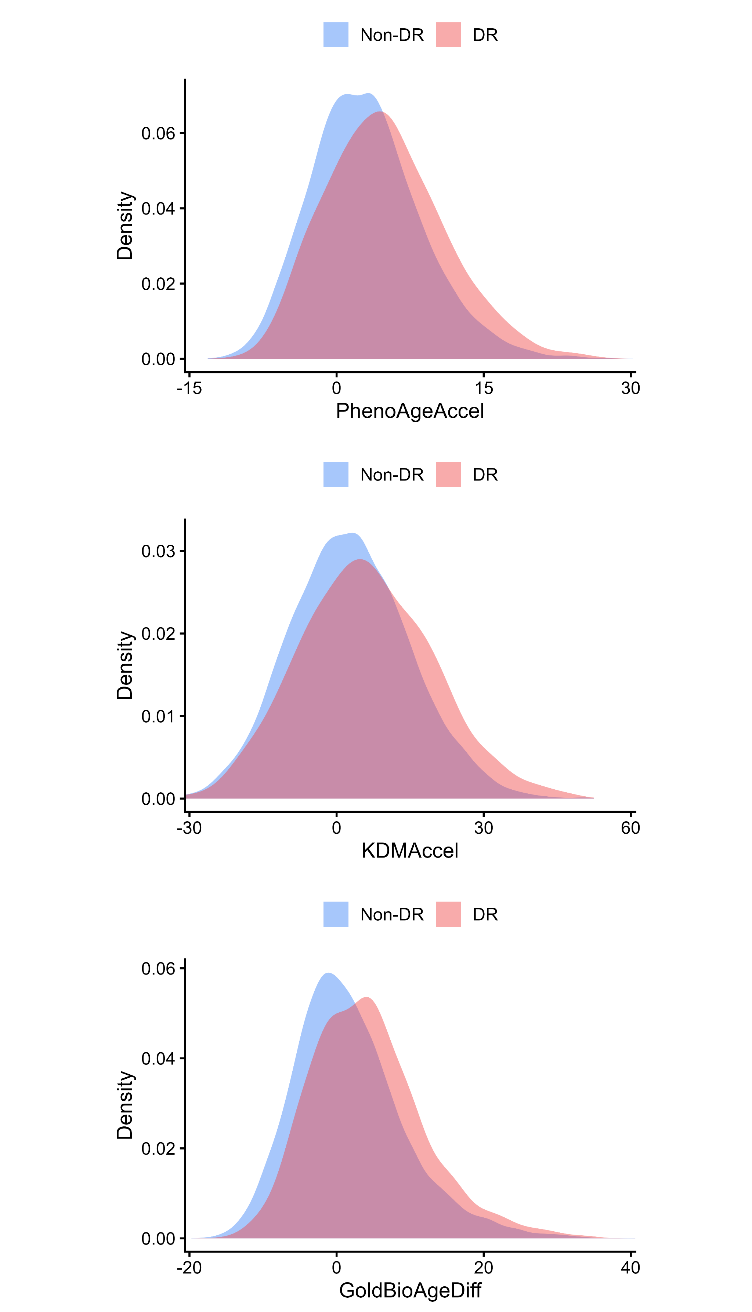


Figure S2. Distributions of three biological age acceleration metrics by DR status among participants with diabetes. DR: diabetic retinopathy; PhenoAgeAccel: phenotypic age acceleration; KDMAccel: Klemera-Doubal method Biological Age acceleration; GOLDBioAgeDiff: Gompertz Law-Based Biological Age difference.


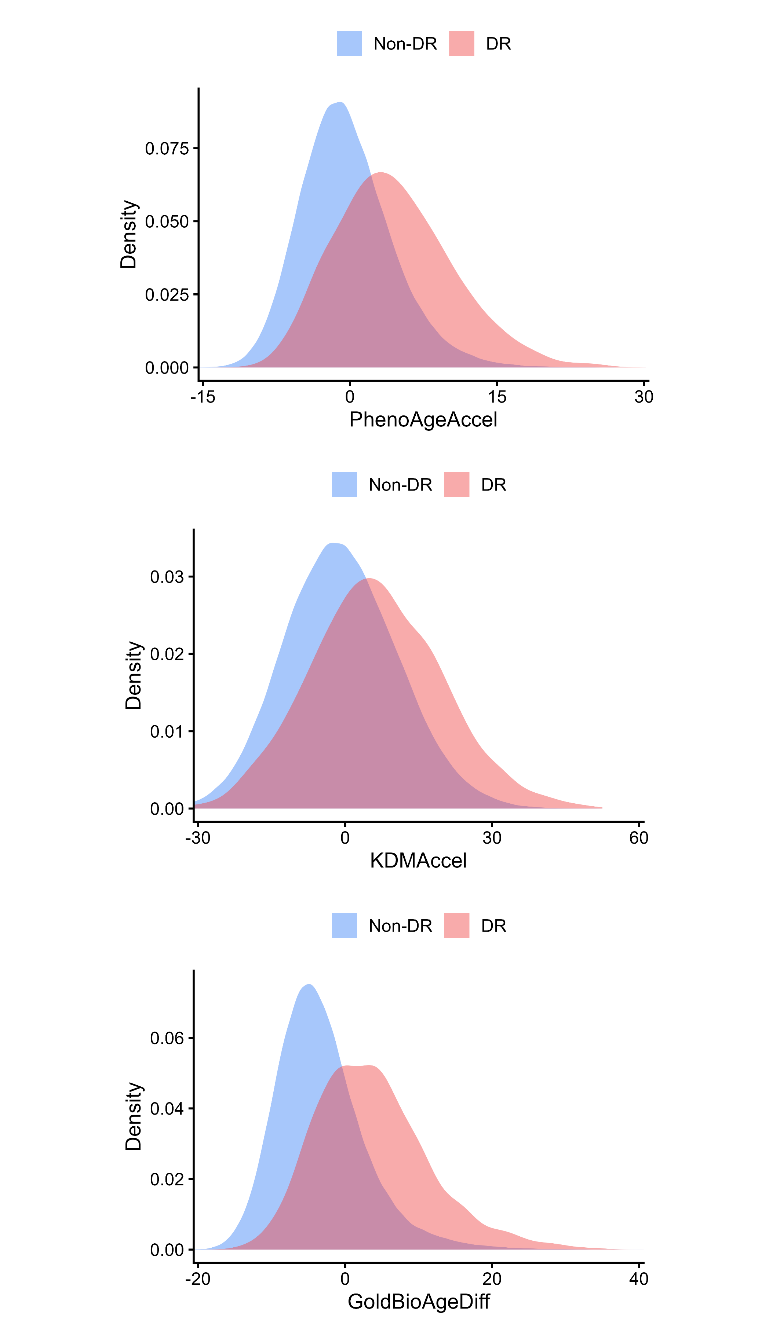


Figure S3. Distributions of three biological age acceleration metrics by DR status in overall population. DR: diabetic retinopathy; PhenoAgeAccel: phenotypic age acceleration; KDMAccel: Klemera-Doubal method Biological Age acceleration; GOLDBioAgeDiff: Gompertz Law-Based Biological Age difference.


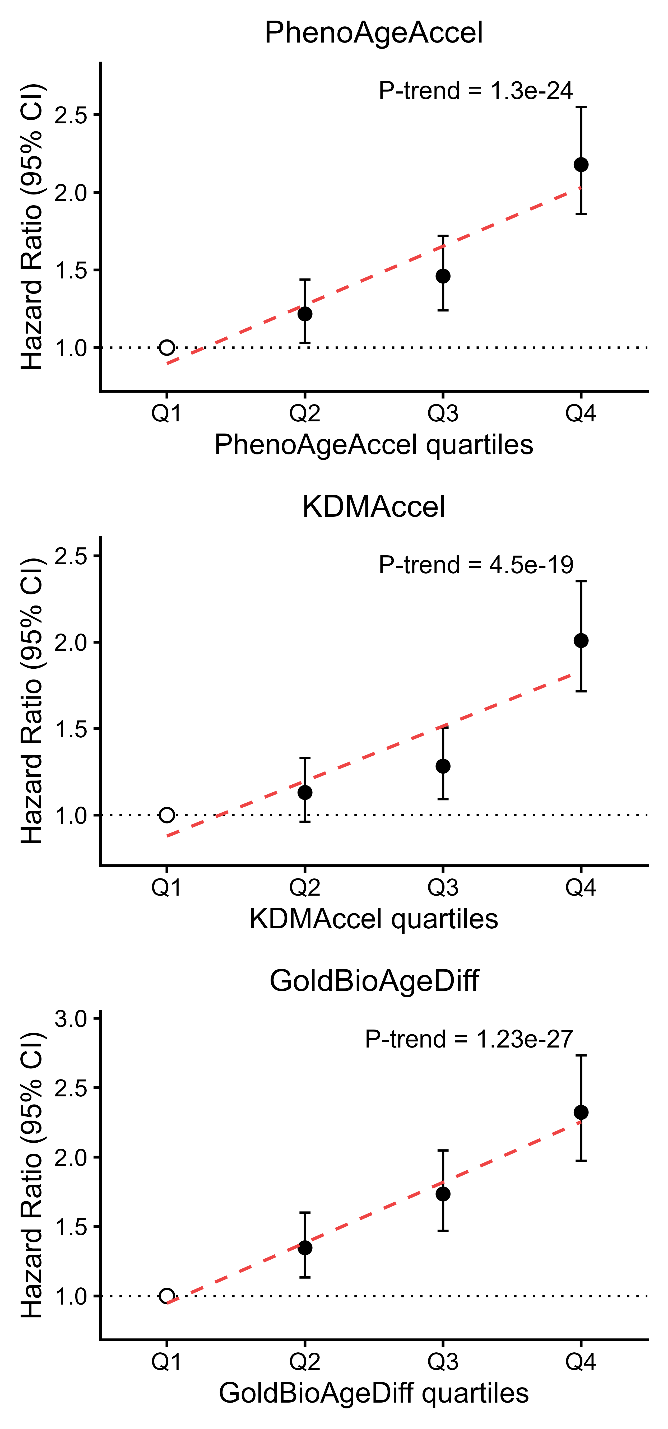


Figure S4. Associations of biological age acceleration quartiles with incident DR risk among participants with diabetes. Chronological age, sex, ethnicity, education, BMI, and Townsend deprivation index, physical activity, sleep duration, smoking, alcohol consumption, and healthy diet were adjusted in the analyses. DR: diabetic retinopathy; PhenoAgeAccel: phenotypic age acceleration; KDMAccel: Klemera-Doubal method Biological Age acceleration; GOLDBioAgeDiff: Gompertz Law-Based Biological Age difference.


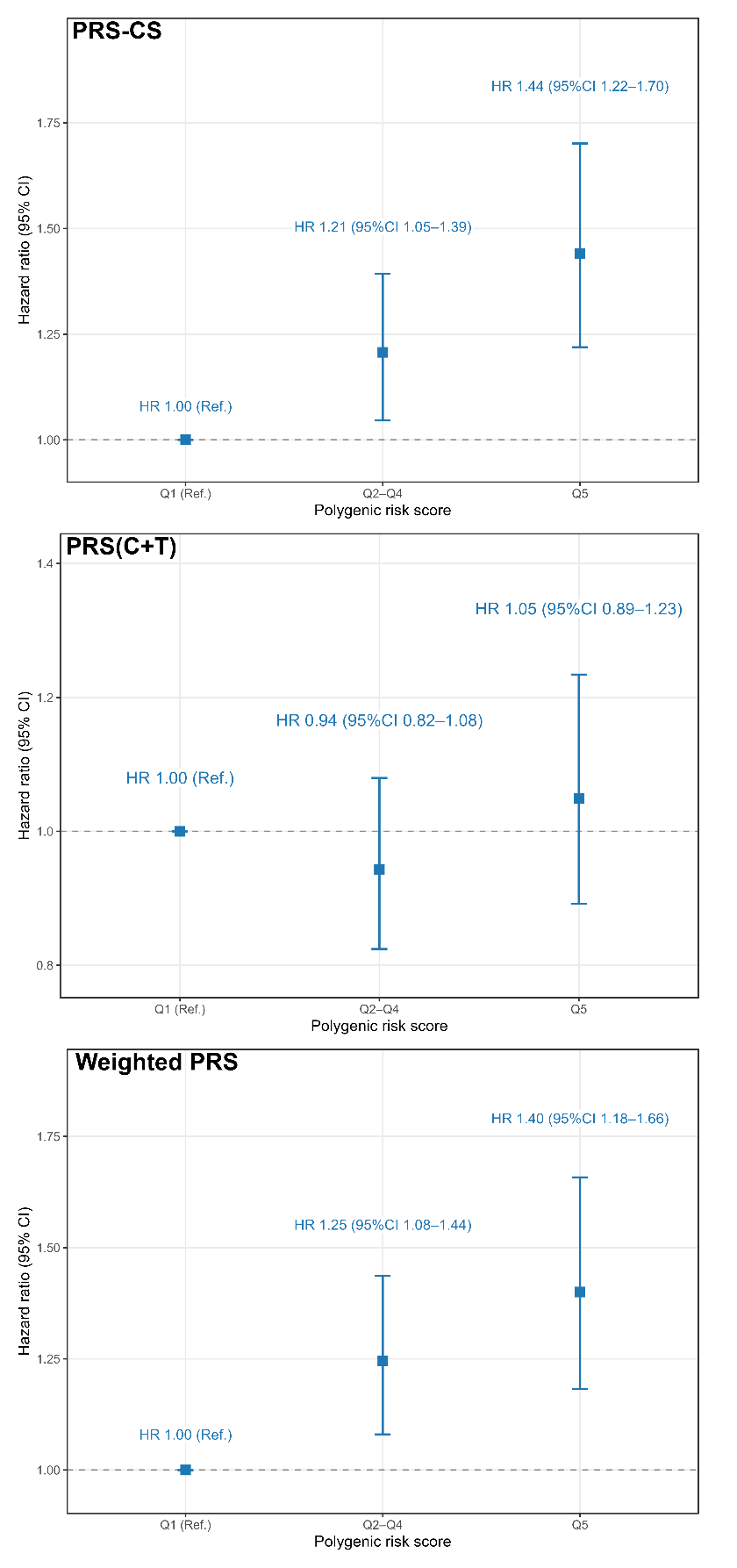


Figure S5. PRS and incident DR risk: grouped quintiles among participants with diabetes (Q1 as reference). Chronological age, sex, ethnicity, education, BMI, and Townsend deprivation index, physical activity, sleep duration, smoking, alcohol consumption, and healthy diet were adjusted in the analyses. PRS: polygenic risk score; DR: diabetic retinopathy.


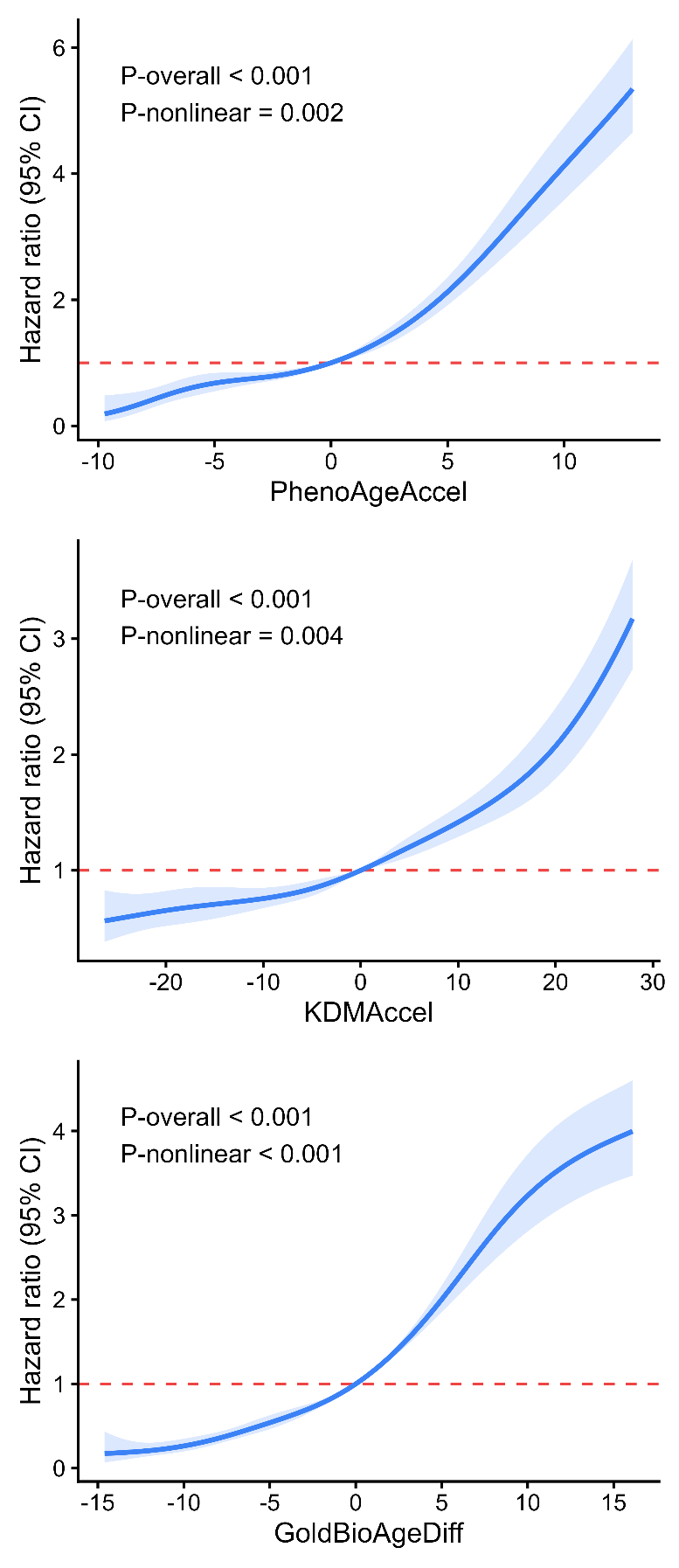


Figure S6. Linear and non-linear association between biological age acceleration and incident DR in overall population. Chronological age, sex, ethnicity, education, BMI, and Townsend deprivation index, physical activity, sleep duration, smoking, alcohol consumption, and healthy diet were adjusted in the RCS analyses. PhenoAgeAccel: phenotypic age acceleration, KDMAccel: Klemera-Doubal method Biological Age acceleration. GOLD BioAgeDiff: Gompertz Law-Based Biological Age difference; DR: diabetic retinopathy.


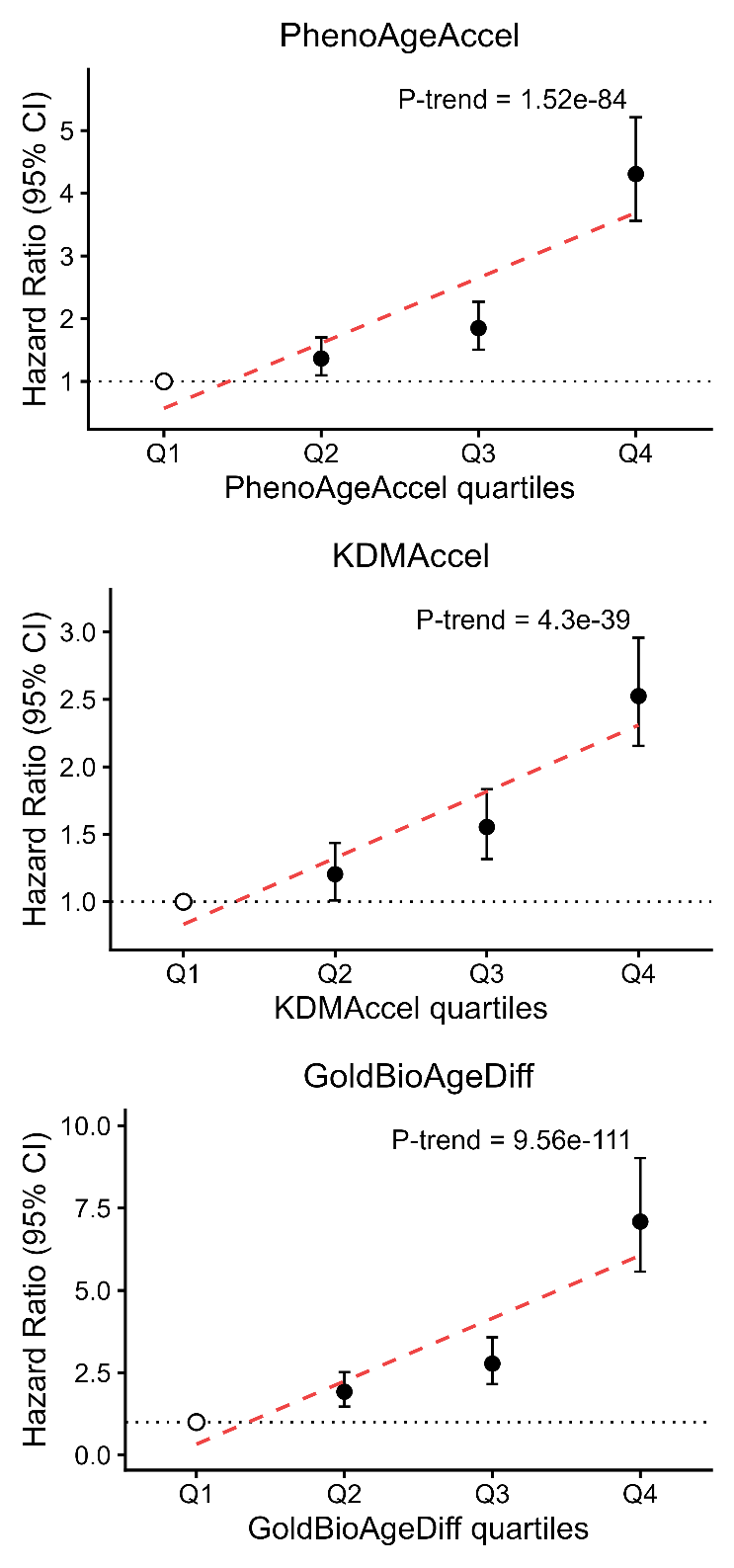


Figure S7. Associations of biological age acceleration quartiles with incident DR risk in overall population. Chronological age, sex, ethnicity, education, BMI, and Townsend deprivation index, physical activity, sleep duration, smoking, alcohol consumption, and healthy diet were adjusted in the analyses. DR: diabetic retinopathy; PhenoAgeAccel: phenotypic age acceleration; KDMAccel: Klemera-Doubal method Biological Age acceleration; GOLDBioAgeDiff: Gompertz Law-Based Biological Age difference.


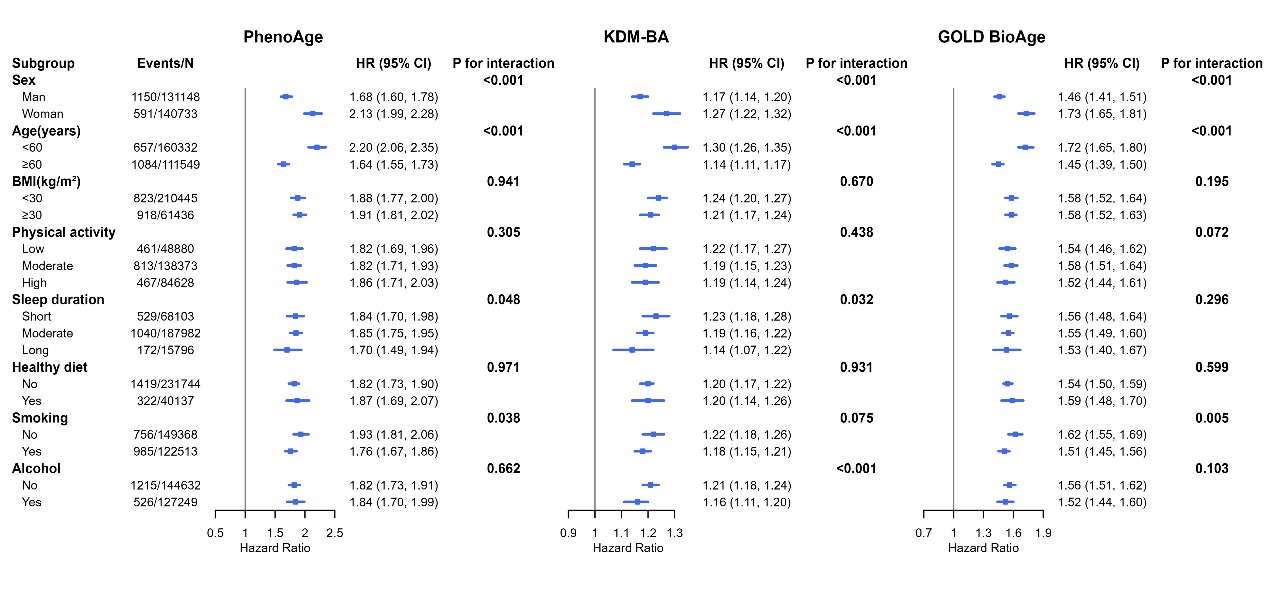


Figure S8. Associations between biological age acceleration and DR risk by different covariates in overall population. Chronological age, sex, ethnicity, education, BMI, and Townsend deprivation index, physical activity, sleep duration, smoking, alcohol consumption, and healthy diet were adjusted in the subgroup analyses. BMI: body mass index; PhenoAge: phenotypic age; KDM-BA: Klemera-Doubal method Biological Age; GOLD BioAgeDiff: Gompertz Law-Based Biological Age difference; DR: diabetic retinopathy.


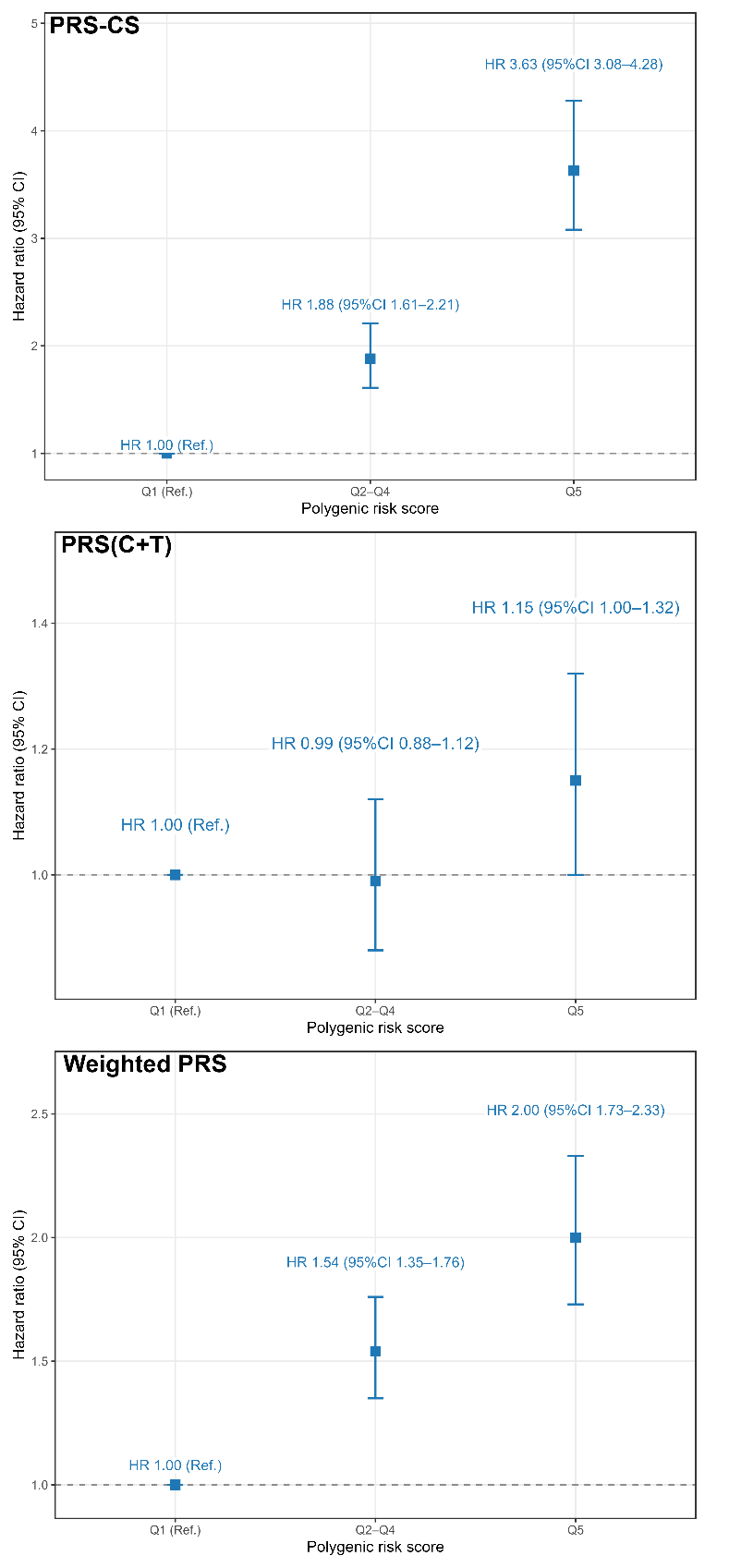


Figure S9. PRS and incident DR risk in overall population: grouped quintiles (Q1 as reference). Chronological age, sex, ethnicity, education, BMI, and Townsend deprivation index, physical activity, sleep duration, smoking, alcohol consumption, and healthy diet were adjusted in the analyses. PRS: polygenic risk score; DR: diabetic retinopathy.

Table S1. Summary of reported susceptibility loci used to construct the weighted PRS.

| RSID | CHR | BP | Effect | Weight |
| --- | --- | --- | --- | --- |
| rs11711732 | 3 | 131382778 | T | 3.407915E-04 |
| rs11916885 | 3 | 186914144 | A | 4.304244E-03 |
| rs6448475 | 4 | 26831827 | T | -7.131335E-04 |
| rs16896723 | 5 | 66701863 | A | 3.348692E-03 |
| rs17109807 | 5 | 148600845 | T | 4.532326E-03 |
| rs201018 | 6 | 6666844 | C | 4.391878E-03 |
| rs2596473 | 6 | 31430799 | T | -2.890713E-04 |
| rs2855812 | 6 | 31472720 | T | 3.607379E-02 |
| rs2857602 | 6 | 31533378 | A | 1.245447E-03 |
| rs2736173 | 6 | 31589735 | T | 1.460575E-02 |
| rs2736157 | 6 | 31600820 | G | 8.328016E-04 |
| rs3117583 | 6 | 31619576 | G | 4.019680E-03 |
| rs652888 | 6 | 31851234 | G | 1.272218E-02 |
| rs1150755 | 6 | 32038550 | T | 1.632588E-02 |
| rs915894 | 6 | 32190390 | G | 2.429392E-02 |
| rs9501398 | 6 | 32202597 | T | 4.560912E-03 |
| rs3129871 | 6 | 32406342 | C | 9.930176E-05 |
| rs3129716 | 6 | 32657436 | C | 7.496073E-02 |
| rs3957146 | 6 | 32681530 | C | 4.266301E-01 |
| rs3998159 | 6 | 32682019 | C | 2.368265E-02 |
| rs9275614 | 6 | 32684257 | G | 1.220157E-02 |
| rs11969718 | 6 | 156286386 | A | 2.292008E-03 |
| rs12698637 | 7 | 67420743 | G | -4.253788E-03 |
| rs4730074 | 7 | 104892139 | C | -4.551940E-03 |
| rs17289756 | 7 | 105712320 | A | 2.121555E-03 |
| rs17066956 | 8 | 3555657 | T | 5.454997E-03 |
| rs2205262 | 8 | 117011884 | A | 6.640552E-04 |
| rs10811661 | 9 | 22134094 | C | -4.011745E-02 |
| rs12684650 | 9 | 139110654 | T | 9.343184E-04 |
| rs12243326 | 10 | 114788815 | C | 2.142957E-02 |
| rs12255372 | 10 | 114808902 | T | 8.832708E-03 |
| rs3842752 | 11 | 2181073 | A | -2.186836E-04 |
| rs16917237 | 11 | 27702383 | T | -8.388265E-04 |
| rs12422853 | 12 | 105063882 | A | 2.816880E-04 |
| rs10132309 | 14 | 101464984 | T | -1.184163E-05 |
| rs7501939 | 17 | 36101156 | C | -2.108794E-02 |
| rs1788234 | 18 | 67566587 | T | 1.726682E-03 |
| rs6046214 | 20 | 19613399 | G | -1.755440E-03 |
| rs138628 | 22 | 44988209 | C | -7.945212E-04 |

RSID: variant identifier; CHR: chromosome; BP: base-pair position; Effect: effect allele used for scoring; Weight: per-SNP weight in the PRS; PRS: polygenic risk score.

Table S2. Coding and definition information of variable in the UK Biobank.

| Variables | Definition | Field ID in UK Biobank |
| --- | --- | --- |
| Diabetic retinopathy | No; Yes | ICD10-H36.0 (41270); ICD9-3620 (41271) |
| Chronological age | Age (years) | Age at recruitment (21022) |
| Sex | Female; Male | Sex (31) |
| Ethnicity | White; Mixed; Asian; Black; Others | Ethnic background (21000) |
| Education level | Degree level or professional education; Other levels | Qualifications (6138) |
| BMI | Body mass index (kg/m^2^) | Body mass index (21001) |
| TDI | Townsend deprivation index | Townsend deprivation index at recruitment (22189) |
| Physical activity | Low; Moderate; High | Summed MET minutes per week for all activity (22040) |
| Sleep duration | Short; Moderate; Long | Sleep duration (1160) |
| Healthy diet | No; Yes | Fresh fruit intake (1309); Dried fruit intake (1319); Cooked vegetable intake (1289); Salad / raw vegetable intake (1299); Bread intake (1438); Bread type (1448); Cereal intake (1458); Cereal type (1468); Oily fish intake (1329); Non-oily fish intake (1339); Cheese intake (1408); Milk type used (1418); Spread type (1428); Non-butter spread type details (2654); Processed meat intake (1439); Age when last ate meat (3680); Poultry intake (1359); Beef intake (1369); Lamb/mutton intake (1379); Pork intake (1389); Never eat eggs, dairy, wheat, sugar (6144) |
| Smoking | No; Yes | Current tobacco smoking (1239); Past tobacco smoking (1249); Light smokers, at least 100 smokes in lifetime (2644) |
| Alcohol | No; Yes | Alcohol intake frequency (1558); Average weekly red wine intake (1568); Average weekly champagne plus white wine intake (1578); Average weekly beer plus cider intake (1588); Average weekly spirits intake (1598); Average weekly fortified wine intake (1608); Average weekly intake of other alcoholic drinks (5364) |
| Albumin | Albumin (g/L) | Albumin (30600) |
| Creatinine | Creatinine (mg/dL) | Creatinine (30700) |
| Glucose serum | Glucose serum (mmol/L) | Glucose (30740) |
| C-reactive protein | C-reactive protein (mg/dL) | C-reactive protein (30710) |
| Lymphocyte percent | Lymphocyte percent (%) | Lymphocyte percentage (30180) |
| Mean cell volume | Mean cell volume (fL) | Mean sphered cell volume (30270) |
| Red cell distribution width | Red cell distribution width (%) | Red blood cell (erythrocyte) distribution width (30070) |
| White blood cell count | White blood cell count (1000 cells/μL) | White blood cell (leukocyte) count (30000) |
| Alkaline phosphatase | Alkaline phosphatase (U/L) | Alkaline phosphatase (30610) |
| Forced expiratory volume in 1 second | Forced expiratory volume in 1 second (L) | Forced expiratory volume in 1-second (FEV1, 3063) |
| Systolic blood pressure | Systolic blood pressure (mmHg) | Systolic blood pressure, automated reading (4080); Systolic blood pressure, manual reading (93) |
| Total cholesterol | Total cholesterol (mg/dL) | Cholesterol (30690) |
| Glycated hemoglobin | Glycated hemoglobin (%) | Glycated hemoglobin (HbA1c, 30750) |
| Urea nitrogen | Urea nitrogen (mg/dL) | Urea (30670) |
| Gamma glutamyl transferase | Gamma glutamyl transferase (U/L) | Gamma glutamyl transferase (30730) |

ICD: International Classification of Diseases.

Table S3. Baseline characteristics in overall population.

|  | Total | Without DR | With DR |
| --- | --- | --- | --- |
| Chronological age (years) | 56.08 ± 8.12 | 56.05 ± 8.12 | 60.19 ± 6.91 |
| Sex |  |  |  |
| Man | 131,148 (48.2%) | 129,998 (48.1%) | 1,150 (66.1%) |
| Woman | 140,733 (51.8%) | 140,142 (51.9%) | 591 (33.9%) |
| Ethnicity |  |  |  |
| White | 259,299 (95.4%) | 257,781 (95.4%) | 1,518 (87.2%) |
| Mixed | 1,504 (0.6%) | 1,493 (0.6%) | 11 (0.6%) |
| Asian | 4,328 (1.6%) | 4,223 (1.6%) | 105 (6.0%) |
| Black | 3,140 (1.2%) | 3,077 (1.1%) | 63 (3.6%) |
| Others | 3,610 (1.3%) | 3,566 (1.3%) | 44 (2.5%) |
| Education level |  |  |  |
| Degree level or professional education | 97,822 (36.0%) | 97,349 (36.0%) | 473 (27.2%) |
| Other levels | 174,059 (64.0%) | 172,791 (64.0%) | 1,268 (72.8%) |
| BMI (kg/m^2^) | 27.20 ± 4.60 | 27.18 ± 4.58 | 31.18 ± 5.74 |
| Townsend deprivation index | -1.48 ± 2.99 | -1.49 ± 2.99 | -0.62 ± 3.37 |
| Physical activity |  |  |  |
| Low | 48,880 (18.0%) | 48,419 (17.9%) | 461 (26.5%) |
| Moderate | 138,373 (50.9%) | 137,560 (50.9%) | 813 (46.7%) |
| High | 84,628 (31.1%) | 84,161 (31.2%) | 467 (26.8%) |
| Sleep duration |  |  |  |
| Short | 68,103 (25.0%) | 67,574 (25.0%) | 529 (30.4%) |
| Moderate | 187,982 (69.1%) | 186,942 (69.2%) | 1,040 (59.7%) |
| Long | 15,796 (5.8%) | 15,624 (5.8%) | 172 (9.9%) |
| Healthy diet |  |  |  |
| No | 231,744 (85.2%) | 230,325 (85.3%) | 1,419 (81.5%) |
| Yes | 40,137 (14.8%) | 39,815 (14.7%) | 322 (18.5%) |
| Smoking |  |  |  |
| No | 149,368 (54.9%) | 148,612 (55.0%) | 756 (43.4%) |
| Yes | 122,513 (45.1%) | 121,528 (45.0%) | 985 (56.6%) |
| Alcohol |  |  |  |
| No | 144,632 (53.2%) | 143,417 (53.1%) | 1,215 (69.8%) |
| Yes | 127,249 (46.8%) | 126,723 (46.9%) | 526 (30.2%) |
| Albumin (g/L) | 45.31 ± 2.52 | 45.32 ± 2.52 | 44.59 ± 2.78 |
| Creatinine (mg/dL) | 72.33 ± 14.15 | 72.31 ± 14.12 | 75.33 ± 18.01 |
| Glucose serum (mmol/L) | 5.06 ± 0.92 | 5.05 ± 0.88 | 7.62 ± 2.31 |
| C-reactive protein (mmol/L) | 0.23 ± 0.32 | 0.23 ± 0.32 | 0.33 ± 0.41 |
| Lymphocyte percent (%) | 28.96 ± 7.10 | 28.97 ± 7.09 | 27.71 ± 7.58 |
| Mean cell volume (fL) | 82.81 ± 5.05 | 82.82 ± 5.05 | 81.56 ± 5.41 |
| Red cell distribution width (%) | 13.44 ± 0.84 | 13.44 ± 0.84 | 13.69 ± 1.00 |
| White blood cell count (1000 cells/μL) | 6.79 ± 1.68 | 6.79 ± 1.67 | 7.50 ± 1.86 |
| Alkaline phosphatase (U/L) | 82.00 ± 22.21 | 81.95 ± 22.17 | 89.65 ± 25.62 |
| Forced expiratory volume in 1 second (mL) | 2,784.62 ± 764.60 | 2,786.38 ± 764.53 | 2,510.97 ± 726.18 |
| Systolic blood pressure (mmHg) | 137.28 ± 18.01 | 137.24 ± 18.01 | 143.26 ± 17.29 |
| Total cholesterol (mg/dL) | 220.43 ± 42.68 | 220.69 ± 42.56 | 179.49 ± 42.15 |
| Glycated hemoglobin (%) | 5.41 ± 0.49 | 5.40 ± 0.47 | 7.01 ± 0.86 |
| Urea nitrogen (mg/dL) | 15.06 ± 3.51 | 15.05 ± 3.51 | 16.47 ± 4.37 |
| Gamma glutamyl transferase (U/L) | 35.60 ± 29.98 | 35.52 ± 29.89 | 48.76 ± 39.77 |
| PhenoAge | 45.59 ± 9.67 | 45.54 ± 9.65 | 54.69 ± 8.56 |
| PhenoAgeAccel | -0.31 ± 4.73 | -0.35 ± 4.71 | 4.51 ± 5.97 |
| KDM-BA | 44.86 ± 14.46 | 44.79 ± 14.44 | 56.24 ± 13.67 |
| KDMAccel | -0.67 ± 11.66 | -0.72 ± 11.63 | 6.35 ± 13.40 |
| GOLD BioAge | 52.84 ± 11.10 | 52.76 ± 11.07 | 63.92 ± 10.23 |
| GOLD BioAgeDiff | -3.25 ± 6.17 | -3.29 ± 6.13 | 3.74 ± 7.76 |

Values are presented as mean±SD or n (%). FEV1: forced expiratory volume in 1 second; BMI: body mass index; DR: diabetic retinopathy; PhenoAge: phenotypic age; KDM-BA: Klemera-Doubal method Biological Age; GOLD BioAgeDiff: Gompertz Law-Based Biological Age difference; PhenoAgeAccel: phenotypic age acceleration, KDMAccel: Klemera-Doubal method Biological Age acceleration.

Table S4. Additive interaction between biological age acceleration and PRS for incident DR among participants with diabetes.

| Category | Additive interaction | | | |
| --- | --- | --- | --- | --- |
|  | Intermediate PRS | | High PRS | |
|  | RERI (95%CI) | AP (95%CI) | RERI (95%CI) | AP (95%CI) |
| PhenoAge | | | | |
| Biological older | 0.28 (-0.13–0.61) | 0.16 (-0.06–0.38) | 0.36 (-0.16–0.83) | 0.17 (-0.07–0.39) |
| KDM-BA | | | | |
| Biological older | 0.31 (-0.05–0.59) | 0.19 (-0.03–0.40) | 0.64 (0.21–1.02) | 0.32 (0.10–0.51) |
| GOLD BioAge | | | | |
| Biological older | 0.10 (-0.37–0.44) | 0.04 (-0.16–0.22) | 0.12 (-0.46–0.62) | 0.05 (-0.18–0.24) |

Values are the RERI and the AP with 95% CIs for biologically older vs. younger within strata of PRS. Chronological age, sex, ethnicity, education, BMI, and Townsend deprivation index, physical activity, sleep duration, smoking, alcohol consumption, and healthy diet were adjusted in the analyses. PRS: polygenic risk score; DR: diabetic retinopathy; RERI: relative excess risk due to interaction; AP: attributable proportion.

| Exposures | Events/Total | Model 1 | | Model 2 | |
| --- | --- | --- | --- | --- | --- |
|  |  | HR (95% CI) | P-value | HR (95% CI) | P-value |
| PhenoAgeAccel (continuous) | | | | | |
| Per 5 years increase | 1741/271881 | 1.86 (1.78–1.93) | <0.001 | 1.82 (1.75–1.90) | <0.001 |
| PhenoAgeAccel (Category) | | | | | |
| Biologically younger | 405/153350 | 1.00 (reference) |  | 1.00 (reference) |  |
| Biologically older | 1336/118531 | 2.78 (2.47–3.12) | <0.001 | 2.70 (2.41–3.04) | <0.001 |
| PhenoAgeAccel (quartile) | | | | | |
| Q1 (<-3.58) | 127/67971 | 1.00 (reference) |  | 1.00 (reference) |  |
| Q2 (-3.58–-0.72) | 216/67970 | 1.37 (1.10–1.70) | 0.006 | 1.37 (1.10–1.70) | 0.006 |
| Q3 (-0.72–2.49) | 344/67970 | 1.86 (1.51–2.28) | <0.001 | 1.85 (1.51–2.27) | <0.001 |
| Q4 (>2.49) | 1054/67970 | 4.45 (3.68–5.38) | <0.001 | 4.31 (3.56–5.21) | <0.001 |
| KDMAccel (continuous) | | | | | |
| Per 5 years increase | 1741/271881 | 1.20 (1.17–1.22) | <0.001 | 1.20 (1.17–1.22) | <0.001 |
| KDMAccel (category) | | | | | |
| Biologically younger | 557/145734 | 1.00 (reference) |  | 1.00 (reference) |  |
| Biologically older | 1184/126147 | 1.83 (1.65–2.04) | <0.001 | 1.84 (1.65–2.04) | <0.001 |
| KDMAccel (quartile) | | | | | |
| Q1 (<-8.75) | 224/67971 | 1.00 (reference) |  | 1.00 (reference) |  |
| Q2 (-8.75–-1.05) | 284/67970 | 1.19 (1.00–1.42) | 0.049 | 1.20 (1.01–1.44) | 0.039 |
| Q3 (-1.05–7.02) | 411/67970 | 1.53 (1.30–1.81) | <0.001 | 1.55 (1.32–1.83) | <0.001 |
| Q4 (>7.02) | 822/67970 | 2.51 (2.14–2.94) | <0.001 | 2.52 (2.16–2.96) | <0.001 |
| GOLD BioAge acceleration (continuous) | | | | | |
| Per 5 years increase | 1741/271881 | 1.54 (1.50–1.58) | <0.001 | 1.55 (1.51–1.59) | <0.001 |
| GOLD BioAge acceleration (category) | | | | | |
| Biologically younger | 602/204155 | 1.00 (reference) |  | 1.00 (reference) |  |
| Biologically older | 1139/67726 | 3.39 (3.06–3.76) | <0.001 | 3.39 (3.06–3.77) | <0.001 |
| GOLD BioAge acceleration (quartile) | | | | | |
| Q1 (<-7.42) | 74/67971 | 1.00 (reference) |  | 1.00 (reference) |  |
| Q2 (-7.42–-3.99) | 189/67970 | 1.91 (1.46–2.51) | <0.001 | 1.92 (1.47–2.52) | <0.001 |
| Q3 (-3.99–-0.02) | 337/67970 | 2.76 (2.14–3.56) | <0.001 | 2.78 (2.15–3.58) | <0.001 |
| Q4 (>-0.02) | 1141/67970 | 7.05 (5.54–8.97) | <0.001 | 7.09 (5.57–9.02) | <0.001 |

Table S5. Association between biological age acceleration and DR risk in overall population.

Model 1 adjusted for chronological age, sex, ethnicity, education, BMI, and Townsend deprivation index. Model 2 further adjusted for physical activity, sleep duration, smoking, alcohol consumption, and healthy diet. PhenoAgeAccel: phenotypic age acceleration, KDMAccel: Klemera-Doubal method Biological Age acceleration; GOLD BioAgeDiff: Gompertz Law-Based Biological Age difference; DR: diabetic retinopathy.

Table S6. Joint effects of biological age acceleration and genetic risk on DR risk in overall population.

| PhenoAgeAccel | | | | |
| --- | --- | --- | --- | --- |
| Subgroup | Events/Total | Incidence events per  100 000 person-years | HR (95% CI) | P-value |
| Low genetic risk | | | | |
| Biological younger | 33/31849 | 6.35 | 1.00 (reference) |  |
| Biological older | 119/22528 | 32.57 | 3.33 (2.26–4.90) | <0.001 |
| Intermediate genetic risk | | | | |
| Biological younger | 215/91929 | 14.36 | 2.11 (1.47–3.05) | <0.001 |
| Biological older | 730/71199 | 63.41 | 5.92 (4.17–8.40) | <0.001 |
| High genetic risk | | | | |
| Biological younger | 157/29572 | 32.67 | 4.50 (3.09–6.55) | <0.001 |
| Biological older | 487/24804 | 122.02 | 10.75 (7.54–15.32) | <0.001 |
| KDMAccel | | | | |
| Subgroup | Events/Total | Incidence events per  100 000 person-years | HR (95% CI) | P-value |
| Low genetic risk | | | | |
| Biological younger | 51/30849 | 10.16 | 1.00 (reference) |  |
| Biological older | 101/23528 | 26.39 | 1.98 (1.41–2.77) | <0.001 |
| Intermediate genetic risk | | | | |
| Biological younger | 305/87410 | 21.49 | 1.95 (1.45–2.62) | <0.001 |
| Biological older | 640/75718 | 52.09 | 3.58 (2.68–4.77) | <0.001 |
| High genetic risk | | | | |
| Biological younger | 201/27475 | 45.13 | 3.82 (2.81–5.20) | <0.001 |
| Biological older | 443/26901 | 101.98 | 6.60 (4.93–8.85) | <0.001 |
| GOLD BioAgeDiff | | | | |
| Subgroup | Events/Total | Incidence events per  100 000 person-years | HR (95% CI) | P-value |
| Low genetic risk | | | | |
| Biological younger | 48/41671 | 7.07 | 1.00 (reference) |  |
| Biological older | 104/12706 | 50.59 | 4.34 (3.08–6.11) | <0.001 |
| Intermediate genetic risk | | | | |
| Biological younger | 313/122649 | 15.68 | 2.06 (1.52–2.80) | <0.001 |
| Biological older | 632/40479 | 96.88 | 7.54 (5.61–10.13) | <0.001 |
| High genetic risk | | | | |
| Biological younger | 241/39835 | 37.26 | 4.60 (3.37–6.28) | <0.001 |
| Biological older | 403/14541 | 173.03 | 12.73 (9.42–17.22) | <0.001 |

Chronological age, sex, ethnicity, education, BMI, and Townsend deprivation index, physical activity, sleep duration, smoking, alcohol consumption, and healthy diet were adjusted in the analyses. PhenoAgeAccel: phenotypic age acceleration, KDMAccel: Klemera-Doubal method Biological Age acceleration; GOLD BioAgeDiff: Gompertz Law-Based Biological Age difference; DR: diabetic retinopathy.

Table S7. Additive interaction between biological age acceleration and PRS for incident DR in overall population.

| Category | Additive interaction | | | |
| --- | --- | --- | --- | --- |
|  | Intermediate PRS | | High PRS | |
|  | RERI (95%CI) | AP (95%CI) | RERI (95%CI) | AP (95%CI) |
| PhenoAge | | | | |
| Biological older | 1.47 (0.63–2.38) | 0.25 (0.10–0.39) | 3.92 (2.45–6.24) | 0.37 (0.24–0.47) |
| KDM-BA | | | | |
| Biological older | 0.66 (0.08–1.14) | 0.18 (0.02–0.33) | 1.81 (0.92–2.78) | 0.27 (0.14–0.39) |
| GOLD BioAge | | | | |
| Biological older | 2.14 (1.11–3.37) | 0.28 (0.15–0.42) | 4.80 (3.03–7.20) | 0.38 (0.26–0.48) |

Values are the RERI and the AP with 95% CIs for biologically older vs. younger within strata of PRS. Chronological age, sex, ethnicity, education, BMI, and Townsend deprivation index, physical activity, sleep duration, smoking, alcohol consumption, and healthy diet were adjusted in the analyses. PRS: polygenic risk score; DR: diabetic retinopathy; RERI: relative excess risk due to interaction; AP: attributable proportion.
